# A systematic comparison of data- and knowledge-driven approaches to disease subtype discovery

**DOI:** 10.1093/bib/bbab314

**Published:** 2021-08-13

**Authors:** Teemu J Rintala, Antonio Federico, Leena Latonen, Dario Greco, Vittorio Fortino

**Affiliations:** Institute of Biomedicine University of Eastern Finland, Yliopistonranta 1 E, 70210 Kuopio, Finland; Faculty of Medicine and Health Technology Tampere University, Kalevantie, 4 33100 Tampere, Finland; BioMediTech Institute Tampere University, Kalevantie 4, 33100 Tampere, Finland; Institute of Biomedicine University of Eastern Finland, Yliopistonranta 1 E, 70210 Kuopio, Finland; Faculty of Medicine and Health Technology Tampere University, Kalevantie, 4 33100 Tampere, Finland; BioMediTech Institute Tampere University, Kalevantie 4, 33100 Tampere, Finland; Institute of Biotechnology University of Helsinki, Viikinkaari 5d, 00014 Helsinki, Finland; Institute of Biomedicine University of Eastern Finland, Yliopistonranta 1 E, 70210 Kuopio, Finland

**Keywords:** transcriptomics, clustering, pathway enrichment analysis, network analysis, cancer, multi-objective

## Abstract

Typical clustering analysis for large-scale genomics data combines two unsupervised learning techniques: dimensionality reduction and clustering (DR-CL) methods. It has been demonstrated that transforming gene expression to pathway-level information can improve the robustness and interpretability of disease grouping results. This approach, referred to as biological knowledge-driven clustering (BK-CL) approach, is often neglected, due to a lack of tools enabling systematic comparisons with more established DR-based methods. Moreover, classic clustering metrics based on group separability tend to favor the DR-CL paradigm, which may increase the risk of identifying less actionable disease subtypes that have ambiguous biological and clinical explanations. Hence, there is a need for developing metrics that assess biological and clinical relevance. To facilitate the systematic analysis of BK-CL methods, we propose a computational protocol for quantitative analysis of clustering results derived from both DR-CL and BK-CL methods. Moreover, we propose a new BK-CL method that combines prior knowledge of disease relevant genes, network diffusion algorithms and gene set enrichment analysis to generate robust pathway-level information. Benchmarking studies were conducted to compare the grouping results from different DR-CL and BK-CL approaches with respect to standard clustering evaluation metrics, concordance with known subtypes, association with clinical outcomes and disease modules in co-expression networks of genes. No single approach dominated every metric, showing the importance multi-objective evaluation in clustering analysis. However, we demonstrated that, on gene expression data sets derived from TCGA samples, the BK-CL approach can find groupings that provide significant prognostic value in both breast and prostate cancers.

## Introduction

High-throughput sequencing technologies and next generation omics platforms have enabled molecular profiling of disease at an unprecedented level. Transcriptomics data obtained from RNA-Seq have been successfully used to identify clinically relevant subtypes of a number of cancer types, including breast cancer [21], colon cancer [45], glioma [62], melanoma [53] and lung cancer [12]. However, technical noise in omics data can lead to false discoveries and missed genes which can dramatically affect disease subtyping results [37]. Commonly used hierarchical clustering (HC) algorithms can perform well with adequate feature selection via differential expression analysis or any other univariate analysis aiming to find disease-relevant genes. Other common methods such as k-means [42] may not be applicable to high-dimensional data due to the ‘curse of dimensionality’ which states that as the number of dimensions increases data points tend to become sparser with respect to most distance metrics [69]. Computing time is also significantly increased for high dimensional data, which is an issue when systematically testing multiple methods with different hyper-parameter settings. As a result of these two issues, the use of dimensionality reduction (DR) techniques is commonplace when applying clustering algorithms to high-dimensional data. Principal component analysis (PCA) [56], t-SNE [41] and UMAP [8] are popular approaches to reduce high-dimensional transcriptomic data into low-dimensional representations. However, the combined use of DR and clustering methods, which is referred to as DR-CL approach, can still fail to efficiently cluster patients due to the challenges imposed by high-throughput data and its nonlinearity [44]. Integration of prior biological knowledge (BK) and molecular networks can overcome the consequences of technical noise in transcriptomics data and increase the robustness of grouping results. On the other hand, the sources of prior BK could be incomplete, inaccurate or biased toward established subtype classifications.

Pathway-based approaches such as GSVA [22] and DiffRank [63] are attractive to the subtype discovery task since they can transform gene-expression profiles to pathway-based profiles without any prior subgroup definition or additional information other than gene sets or functional annotations. Such profiles can then be used to compile robust patient stratification results [40, 67]. Another interesting approach for pathway-based clustering analysis is integrating (transcript-)omics data with molecular networks to characterize biological pathway activity. For instance, gene co-expression networks are often used to highlight patient-centric subnetworks, while protein–protein interaction (PPI) networks have been successfully used for constructing biological pathways [19]. In contrast to the methods applying gene statistics to profile pathway activity, the network-based approach does not ignore the complex topology of functional relations existing among genes or proteins [4]. These relations can highlight genes that are closely linked to differentially expressed or dysregulated genes but whose signal is otherwise lost in the noisy omics profiles.

Although pathway- and network-based approaches may lead to better patient stratification results in cancer, there is currently no protocol to support a systematic comparative analysis between DR-CL and biological knowledge-driven clustering (BK-CL) approaches. In this study, we aim at filling this gap by providing a computational framework which implements existing DR-CL and BK-CL approaches and a novel network analysis and pathway enrichment method based on a combination of random walk with restart (RWR) and gene set enrichment analysis (GSEA). Disease subtyping techniques are systematically evaluated through different criteria, which include (1) internal evaluation metrics [e.g. silhouette score (SS)]; (2) external evaluation metrics, which are based on association with known disease subtypes and batch effects; (3) functional relevance; and (4) clinical utility. Separability is important for identifying clearly distinct molecular patterns, but assessing the other criteria may be more important in order to obtain more actionable subtyping results. In this study, we decided to apply survival analysis for assessing clinical differences of the clusters. Moreover, we propose a novel disease module-based score which verifies that the clusters correlate with different network-driven gene modules. Many basic machine learning methods have been benchmarked on TCGA data (e.g. [61]). Therefore, we decided to conduct our benchmarking analysis using gene expression data from two TCGA cancer data sets: invasive breast carcinoma (BRCA) and prostate adenocarcinoma (PRAD).

## Materials and methods

### TCGA RNA-Seq data processing

Normalized RNA-Seq data for BRCA and PRAD was downloaded with the *curatedTCGAData* [57] R-package. The retrieved data consisted of upper-quartile-normalized TPM values from the RSEM method [38] which were log-transformed before entering the clustering analysis. Genes with near zero variance were identified and removed by using the *caret*-package [33].

### Selection of gold-standard subtypes BRCA and PRAD

Subtypes and other clinical and technical variables were retrieved with *TCGAbiolinks* [14] and *curatedTCGAData*. The molecular subtypes in BRCA are based on the PAM50 assay [55]. *TCGAbiolinks* PAM50 subtypes are sourced from [10]. Two samples with missing subtype data were excluded. Moreover, we removed 40 patients with the normal-like subtype, which is not as well defined as the Luminal A, Luminal B, Her2 over-expressed and basal-like subtypes [16, 55]. Prostate cancer subtypes are poorly understood in terms of gene expression, although subtypes based on somatic mutations have been identified [28, 54]. Therefore, we used the Gleason score to define subtypes for evaluation in PRAD. More specifically, we used four different Gleason score categories based on primary and secondary Gleason scores: <7, 3+4, 4+3 and >7. The 3+4 and 4+3 categories have been suggested to have different prognoses [68]. The tumor samples were acquired prior to treatment, with the exception of three breast cancer patients who had received prior treatment.

### Selection of disease-relevant genes for BRCA and PRAD

The available tumor-control sample pairs were used to perform a differential expression analysis by using the negative binomial generalized log-linear model method implemented in *TCGAbiolinks*. Differentially expressed genes (DEGs) were identified by BH-adjusted [9] $P<$$0.01$ and log-fold change greater than $1.0$. Open Targets platform (OTP) [32] was used to retrieve known disease-gene associations which we intersected with the DEGs to obtain a small set of highly relevant genes for each cancer type. OTP provides scores based on multiple evidence categories and we primarily used the genetic association score which is based on genome wide association studies (GWASs). We found that the intersection of DEGs and OTP genes yielded the best results from BK-CL approaches; hence, the presented results (including results for DR-CL) will use the intersection unless indicated otherwise. The set of genetically associated genes for PRAD in the OTP database had low overlap with DEGs and yielded poor results for pathway enrichment-based workflows, which is why the overall association score was used for PRAD instead. Supplementary Tables S1 and S2 provide a summary and numerical detail of the data used.

### Dimensionality reduction methods

Gene expression data was transformed into low-dimensional representations by using various dimensionality reduction techniques in order to efficiently test different clustering algorithms. We used four different methods: PCA [56], t-SNE [41], UMAP [8] and the Variational Autoencoder (VAE) [31]. PCA, t-SNE and UMAP are commonly used in exploratory data analysis. PCA factorizes data into linear components while the latter two are based on manifold learning and transform the data nonlinearly into a low-dimensional embedding where distances between data points are approximately preserved at the local level which can improve clustering performance [3]. The analysis of dimensionality reduction results can lead to selecting different number of dimensions. We evaluated all clustering results for two to ten dimensions for both PCA and UMAP. We then chose the best number of dimensions based on the average of the sum of SS, stability, batch effect, functional relevance and clinical relevance across different clustering methods and different number of clusters. Two principal components were selected for both BRCA and PRAD, while five latent UMAP dimensions were selected for BRCA and two for PRAD. On the other hand, t-SNE only supports two or three dimensions [41] and two were used for both data sets. We also tested different hyper-parameter values for t-SNE perplexity and UMAP number of neighbors. For t-SNE perplexity 45 was found to be the most consistent value between 15 and 45 that we tested, while 20 was the most consistent number of neighbors for UMAP based on testing values between 10 and 30. We also tested a VAE which is a variational-Bayes extension of autoencoders. The autoencoder is an unsupervised deep learning method that uses multiple layers of nonlinear functions to transform input data into a low-dimensional representation and then aims to reconstruct the original data from this embedding. The VAE embedding is probabilistic which could help address the heterogeneity of cancer data [65]. A detailed description of the VAE-based approach can be found in the Supplementary Methods and Results file.

### Pathway enrichment

GSVA [22] and DiffRank [63] were used to transform the gene expression profiles of each sample into pathway enrichment scores. These methods are based on gene statistics that are computed for each sample based on the empirical distribution of each gene within samples. They summarize the statistics of gene sets derived from functional annotations in order to estimate the activity of the corresponding pathway with a single value. Functional annotations including KEGG pathways [29], REACTOME pathways [26] and Gene Ontology biological process (GO-BP) terms [6] and cancer hallmark gene sets were retrieved from MSigDB version 7.2 by using the msigdbr-package [39].

### A network-driven pathway enrichment approach for feature extraction in omics datasets

Next, we outline a novel topology enhanced pathway enrichment method for patient stratification. Supplementary Figure S1 illustrates the workflow of the implemented approach which takes as input a gene expression matrix, a gene-network as well as functional annotations, i.e. gene sets. As described above, we first identify a small set of relevant genes that are differentially expressed between tumor-control pairs and identified as disease-associated in the OTP database. The genes are split into up- and down-regulated lists based on the sign of the fold change. Genes in each list are ranked based on the gene expression profiles of each patient sample. Then, in order to define patient-driven sets of dysregulated genes, the top-ranked genes are selected in both up- and down-regulated gene lists. In the second part of the proposed pipeline, the patient-based gene sets are utilized as starting points (i.e. seeds) of a RWR procedure. RWR is a type of network diffusion algorithm that can be used to boost the discovery of disease-relevant genes [49]. The resulting gene affinities, i.e. the probabilities corresponding to the gene network RWR stationary distribution, correspond to extended sets of dysregulated genes and are finally given in input to the fast pre-ranked GSEA (FGSEA) method in order to determine significantly enriched pathways and to generate normalized enrichment scores (NESs) [60]. Next, NESs were multiplied by the logarithm of the corresponding $P$-values to produce sparse embeddings. These adjusted pathway enrichment scores were finally used as patient-based features in the downstream clustering analysis. In the RWR-FGSEA approach, the number of top-ranked genes to be selected from patient-driven gene rankings was systematically tuned by considering different cut-offs (between 10% and 50% of ranked genes). The best results were obtained by using 50 top-ranked genes, corresponding to approximately 30% of the intersection of differentially expressed and disease-associated genes, for both BRCA and PRAD data sets. The *dnet*-package [17] was used to implement RWR. In more detail, restart probability was set to $0.75$ and a normalized Laplacian matrix was generated from the adjacency matrix and used to compute the random walk affinities. We applied the RWR on both a manually curated PPI network, which can be retrieved from [13], and gene co-expression networks (GCNs) generated from the data by using WGCNA [34]. The retrieved PPI network was directed and unweighted while WGCNA is typically used to generate undirected and weighted networks. However, in order to keep the methods consistent, we used unweighted and undirected networks. For PPI, the directed edges were simply redefined as undirected, and for GCNs, we used the hard-threshold WGCNA method. More specifically, a threshold value was applied to absolute values of Spearman correlation between genes to yield an unweighted adjacency matrix. The threshold was tuned based on the scale-free topology criterion of the resulting network [34]. For BRCA, we selected $0.475$ as the threshold, while for PRAD, we selected $0.525$.

### Clustering algorithms

The data sets resulting from different feature reduction techniques were then evaluated by means of clustering analysis. In total we tested six different clustering methods: k-means [42]; gaussian mixture model (GMM) [46]; agglomerative HC with average, complete [25] and Ward’s linkage [64]; and divisive HC (DIANA) [30]. Clustering algorithms are very sensitive to the distance metric used, especially when the data are very high dimensional. Hence, we used Euclidean distance in the DR-CL workflow and correlation-based distance (defined as $d=1-\textrm{cor}(x,y)$) in the BK-CL workflow. Since k-means, GMM and HC with Ward’s linkage require the use of Euclidean distance, they were only applied in the DR-CL workflow. The k-means implementation from *ClusterR*-package [51] was used with 100 random initializations based on k-means++ [5]. GMM from *mclust*-package [59] was parameterized with a full unrestricted covariance matrix, which allows the estimation of cluster density in a very flexible manner. However, a regularizing prior was used on the means of the mixture components with shrinkage parameter set to $0.01$ in order to avoid numerical problems. For agglomerative HC we used the *flashClust*-package [35] and for DIANA we used the *cluster*-package [43].

### Evaluation strategy and standard metrics

We assessed clustering performance by using external and internal validation metrics as well as clustering stability. In our benchmarking studies, we used the normalized mutual information (NMI) method to evaluate the agreement of a clustering result with known class labels. NMI was also used to assess the association between the clustering results and known batch effects. Samples may be processed through different protocols, depending on the practices followed by each independent laboratory. Therefore, the variable indicating the different laboratories that processed the patient samples should be considered as a possible source of batch effect [50]. However, all biospecimen corresponding to the samples in TCGA BRCA and PRAD were sequenced at the same laboratory [52, 54]. The source of the biospecimen (i.e. tissue source site) could be relevant due to possible differences in sample preparation and transport to the biospecimen storage. Another batch variable that could be considered is the plate ID which identifies batches of samples that were sequenced at the same time. In general, we want to minimize association with the batch variables while maximizing the association with subtypes. To this end, we applied the cNMI metric which is based on the difference between phenotype or subtype NMI and batch NMI [47]. We used the SS (a.k.a. average silhouette width) to evaluate the internal structure of clustering results. SS evaluates the goodness of a clustering result by measuring the compactness of each cluster (or group) and the separation between different clusters. Well-separated and dense clusters tend to have high scores, while low-density and poorly separated clusters tend to result in lower scores. Clustering stability aims to assess the similarity of clustering solutions obtained by applying the same clustering algorithm on different subsets of the same data set. In this study, we used 20 repeats of 5-fold cross-validation in order to simulate 100 different data sets and compile clustering results within selected folds. Then, several similarity metrics, such as Jaccard similarity [24] and adjusted Rand index (ARI) [23], were used to assess the stability of clustering results.

### Survival analysis

The Cox proportional hazards (PH) model [15] was used to assess differences in patient survival between clustering results. In order to compare survival across different numbers of clusters we used a likelihood ratio test (LRT) between a baseline model and an alternative model that included an additional categorical variable corresponding to cluster membership. The baseline model was fitted using covariates selected from preliminary analysis limited to the clinical data. TCGA BRCA survival data consisted of 979 patients; the average survival rate was 86.7%. Patient age and tumor stage were identified as significant covariates. A total of 89 BRCA patients with follow-up times beyond 3000 days were excluded as outliers. In TCGA PRAD, biochemical recurrence-free survival was considered as the endpoint due to the small number of fatal cases in the data. Biochemical recurrence data were available for 338 patients for whom the average survival rate was 87.9%. Pre-operative prostate-specific antigen (PSA) and tumor N-stage were identified as significant covariates. Forty-seven PRAD patients with follow-up times beyond 2000 days were excluded as outliers. Covariates were selected via a model selection procedure where available clinical variables were initially tested independently by using a Cox-PH model. We only considered clinical variables with fewer than 20% missing values in the public data sets which were age, stage and pathology for BRCA and PSA blood level, age, T-stage, N-stage and Gleason score category for PRAD. Supplementary Tables S3 and S4 list the Akaike information criterion (AIC) [2] and Bayesian information criterion (BIC) [58] in Cox-PH models including relevant subsets of the clinical variables. The lowest $P$-value of coefficients for models corresponding to individual variables are also shown. For BRCA, age and stage were selected as covariates since both AIC and BIC were minimized for the corresponding model and they had low $P$-values independently. For PRAD, AIC and BIC did not agree and of the three lowest $P$-values age had the highest value, which is why PSA and N-stage were used since the corresponding model had close to minimal AIC and BIC and both were significant predictors independently.

### Gene module score

In order to assess the mechanistic relevance of clusters, we defined a score based on gene modules derived from WGCNA. Gene modules for each data set were identified from a weighted gene co-expression network constructed from Spearman correlations between DEGs by using the soft power method [34]. The correlations are raised to a power selected to meet the scale-free topology criterion. Soft power $6$ was selected for BRCA and $4$ was selected for PRAD. The weighted network was used to compute a topological overlap matrix-based similarity for genes which were then hierarchically clustered to yield gene modules and associated ‘eigen-genes’ [34]. We then defined a gene module score based on correlations between cluster indicators and WGCNA module eigen-genes. The purpose of the score is to assess the mechanistic distinction between the clusters.

The module score is defined as

(1) \begin{align*}& \textrm{score} = E\left[\frac{\min{(1,\mathbf{S^{+})}}+\min{(1,\mathbf{S^{-})}}}{\mathbf{S^{+}} + \mathbf{S^{-}}}\right], \end{align*}

where

$$\begin{align*}& \mathbf{S^\pm_{i}} = \sum_j{\delta^\pm\left(\rho(\mathbf{e}^{(i)}, \mathbf{c}^{(j)})\right),} \end{align*}$$

where $\rho $ corresponds to spearman correlation, $\mathbf{e}^{(i)}$ corresponds to the $i$th column of the WGCNA eigen-gene matrix, $\mathbf{c}^{(j)}$ corresponds to the $j$th cluster indicator variable and

$$\begin{align*}& \delta^{-}(x) = \begin{cases} 1 & \mbox{if } x \leq -\alpha \\ 0 & \mbox{otherwise} \end{cases} \end{align*}$$

$$\begin{align*}& \delta^{+}(x) = \begin{cases} 1 & \mbox{if}\ x \geq \alpha \\ 0 & \mbox{otherwise,} \end{cases} \end{align*}$$

where $\alpha $ is a parameter that defines a threshold for counting correlations.

The fraction is undefined when the denominator is zero which occurs when no clusters are strongly correlated to an eigen-gene, in which case we assign a score of $\beta $. When $\beta $ is close to zero, clustering results that do not exhibit differential expression between clusters in each gene module are penalized. For the results presented in this article, we used $\beta =0$ since we wanted to use the score to estimate the extent to which the clusters cover different gene expression modules.

## Results

### Computational workflow

**Figure 1:**
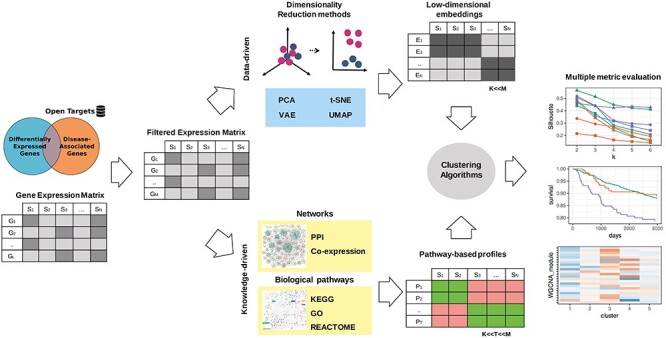
Workflow overview: starting from gene-expression, we transform the data using either data-driven DR methods or BK-based pathway enrichment methods such that we acquire a lower-dimensional representation of the data for each patient. The patients were then clustered based on this representation by using common clustering algorithms and finally evaluated with a common set of metrics. Four different DR methods and three different BK methods were tested. For BK methods, we tested four different gene set collections and two different interaction networks (PPIs and gene co-expression networks).

Figure 1 shows a graphical illustration of the comparative study that we implemented to evaluate DR-CL and BK-CL approaches for disease subtyping. Then, Supplementary Table S5 provides technical details on the methods that were chosen in the present study. The main difference between the two approaches was in how prior BK, represented by disease-relevant genes, pathway annotations and gene networks, was used in BK-CL to reduce the gene expression data into pathway activity profiles corresponding to a reduced set of features while in DR-CL feature reduction was achieved by using computational methods. These reduced profiles were then given as input to clustering algorithms for patient stratification. In order to assess the added value of pathway- and network-driven knowledge, we benchmarked the selected DR-CL and BK-CL methods for patient stratification in two RNA-Seq datasets from TCGA: invasive BRCA and PRAD. The clustering methods were systematically evaluated based on cluster separation, association with currently known subtypes, batch effect, clinical relevance and disease module association. Details on the methods, hyper-parameter settings and biological annotations (including pathway gene sets and the computation of disease-relevant genes) can be found in Materials and Methods. The mean and standard deviation (SD) of metrics for each combination of methods is available given in Supplementary Files S2 and S3. In most cases, the SD was significantly lower than differences between methods and was omitted below for clarity. In the following sections, we provide a summary of the results. A full list of results and the code used to generate them can be found on our GitHub repository (https://github.com/vittoriofortino84/COPS/tree/benchmark).

### Evaluation of internal metrics

Cluster separation and cohesion were evaluated with the SS which is based on the difference in average distance of data points within clusters and between clusters. Tables 1 and 2 show the mean SS for clusters obtained from different approaches applied on TCGA BRCA and PRAD gene expression data sets. For each embedding, the tables only show the best clustering algorithm which was selected based on a sum of SS, clustering stability, batch effect, module relevance and clinical relevance. Well-separated and dense clusters tend to have high SS, while low density and low separation tend to result in lower SS. SS was computed with respect to the Euclidean distance in DR-CL approaches while correlation distance was used in BK-CL SS computation since it was also used in the clustering for these approaches due to the relatively high number of dimensions. In BRCA, for two clusters the UMAP-based approach yielded much higher SSs compared 10with other approaches, which was apparent in visualizations based on UMAP embeddings where the basal subtype was very well separated from the other subtypes (Supplementary Figure S2 and S3). However, the basal-luminal division was retrieved by almost every DR-CL approach, which is why we decided to exclude the two-cluster result from our analysis. Overall, GSVA outperformed DR-CL approaches when using KEGG-based pathways. However, in general, other BK-CL approaches did not show better performances than DR-CL methods. As can be observed, in most cases, average SS decreases as the number of clusters increases. Among DR-CL approaches, VAEs had the lowest SS in BRCA, which is most likely due to the difference in embedding dimensions. In PRAD, the best PCA- and VAE-based results were not far apart in terms of SS even though VAE embeddings had 10 dimensions and PCA had 2. UMAP and t-SNE had similar SS in both cancers, although UMAP was slightly worse than t-SNE in BRCA. However, whether these differences between approaches correspond to differences in the ability to find distinct biological patterns is questionable. The fact is that SS is highly dependent on the number of dimensions (Supplementary Figures S4 and S5). In fact, the effect of dimensions is clearly visible in the BK-CL results as well: KEGG has very few pathways while GO has the most and SS is highest for KEGG while lowest for GO in GSVA and tied for lowest with REACTOME in RWR-FGSEA. An exception to the pattern was the Hallmark collection which has the fewest gene sets, but these gene sets are significantly larger on average, which can result in less pronounced differences in enrichment scores between samples and may have resulted in lower SS clusters in these data sets. Hence, SS should not be used alone for selecting the best groupings.

**Table 1:** Mean of average silhouette width of BRCA clusters for the best clustering algorithm for each different embedding based on the average of the sum of SS, clustering stability, batch effect, module and clinical relevance across different number of clusters (k)

Dimensionality reduction^*^						Dimensionality reduction					
Embedding	Clustering	k = 3	k = 4	k = 5	k = 6	Embedding	Clustering	k = 3	k = 4	k = 5	k = 6
PCA	k-means	0.47	0.40	0.38	0.38	PCA	k-means	0.45	0.39	0.37	0.37
t-SNE	k-means	0.45	0.48	0.46	0.45	t-SNE	k-means	0.47	0.47	0.45	0.43
UMAP	k-means	0.46	0.47	0.47	0.47	UMAP	k-means	0.50	0.49	0.47	0.47
VAE	k-means	0.21	0.20	0.20	0.19	–	–	–	–	–	
DiffRank						GSVA					
Embedding	Clustering	k = 3	k = 4	k = 5	k = 6	Embedding	Clustering	k = 3	k = 4	k = 5	k = 6
GO	DIANA	0.44	0.28	0.25	0.21	GO	HC (avg.)	0.30	0.26	0.24	0.23
KEGG	DIANA	0.29	0.27	0.23	0.19	KEGG	DIANA	0.66	0.62	0.58	0.56
Reactome	DIANA	0.44	0.33	0.30	0.29	Reactome	DIANA	0.49	0.45	0.41	0.37
Hallmark	DIANA	0.35	0.26	0.25	0.22	Hallmark	DIANA	0.31	0.27	0.24	0.22
GCN RWR-FGSEA						PPI RWR-FGSEA					
Embedding	Clustering	k = 3	k = 4	k = 5	k = 6	Embedding	Clustering	k = 3	k = 4	k = 5	k = 6
GO	DIANA	0.32	0.21	0.19	0.17	GO	DIANA	0.30	0.22	0.20	0.18
KEGG	DIANA	0.50	0.38	0.32	0.30	KEGG	HC (avg.)	0.55	0.51	0.52	0.50
Reactome	DIANA	0.26	0.22	0.21	0.20	Reactome	DIANA	0.35	0.27	0.25	0.23
Hallmark	DIANA	0.46	0.45	0.44	0.42	Hallmark	DIANA	0.46	0.43	0.42	0.37

$^{\ast }$ Applied on whole transcriptome (other results were obtained with DEG and disease associated gene intersection).

**Table 2:** Mean of average silhouette width of PRAD clusters for the best clustering algorithm for each different embedding based on the average of the sum of SS, clustering stability, batch effect, module and clinical relevance across different number of clusters (k)

Dimensionality reduction^*^						Dimensionality reduction					
Embedding	Clustering	k = 3	k = 4	k = 5	k = 6	Embedding	Clustering	k = 3	k = 4	k = 5	k = 6
PCA	k-means	0.38	0.34	0.34	0.36	PCA	k-means	0.34	0.36	0.37	0.36
t-SNE	k-means	0.40	0.39	0.38	0.38	t-SNE	k-means	0.39	0.39	0.40	0.39
UMAP	k-means	0.42	0.43	0.43	0.43	UMAP	k-means	0.42	0.43	0.45	0.44
VAE	HC (avg.)	0.28	0.23	0.19	0.16	–	–	–	–	–	
DiffRank						GSVA					
Embedding	Clustering	k = 3	k = 4	k = 5	k = 6	Embedding	Clustering	k = 3	k = 4	k = 5	k = 6
GO	HC (avg.)	0.49	0.43	0.36	0.30	GO	DIANA	0.19	0.16	0.14	0.13
KEGG	DIANA	0.28	0.26	0.24	0.23	KEGG	HC (avg.)	0.68	0.68	0.66	0.66
Reactome	DIANA	0.26	0.24	0.23	0.23	Reactome	DIANA	0.35	0.34	0.31	0.29
Hallmark	DIANA	0.22	0.21	0.20	0.20	Hallmark	DIANA	0.42	0.36	0.34	0.32
GCN RWR-FGSEA						PPI RWR-FGSEA					
Embedding	Clustering	k = 3	k = 4	k = 5	k = 6	Embedding	Clustering	k = 3	k = 4	k = 5	k = 6
GO	DIANA	0.24	0.25	0.24	0.23	GO	DIANA	0.27	0.23	0.22	0.22
KEGG	DIANA	0.42	0.40	0.37	0.30	KEGG	HC (avg.)	0.58	0.55	0.52	0.50
Reactome	DIANA	0.31	0.22	0.20	0.19	Reactome	HC (avg.)	0.37	0.34	0.30	0.27
Hallmark	HC (avg.)	0.56	0.53	0.46	0.42	Hallmark	HC (avg.)	0.32	0.22	0.17	0.15

$^{\ast }$ Applied on whole transcriptome (other results were obtained with DEG and disease associated gene intersection).

On the other hand, clustering stability may be a better metric for comparing different DR-CL and BK-CL approaches, since it does not deteriorate when increasing the number of features. Tables 3 and 4 clustering stability as measured by the Jaccard similarity between the CV-based sample subsets and the reference based on all samples. Similarly to Tables 1 and 2, only the best clustering algorithm for each embedding is shown. PCA and the RWR-based approach were the most stable when applied to different subsets of the data. This is expected as PCA is the only deterministic dimension reduction method that we tested. T-SNE, UMAP and VAE are based on stochastic optimization algorithms and random initialization which resulted in slightly different embeddings every time they were run. GSVA uses an empirical probability distribution which is based on the input samples when computing activity scores, which means that the pathway profile of a given patient depends on the gene-expression of other patients included in the same input data. As a result, GSVA-based clustering results never achieve high stability. In DiffRank, a given profile also depends on the other input samples, but it is rank-based which seems to make it less sensitive to the other samples. RWR-FGSEA is deterministic and does not depend on the other samples since the gene ranking step is performed independently of other samples, which explains the high stability it achieves. Another key factor in achieving highly stable clustering results seems to be the clustering algorithm. DIANA, average linkage HC and k-means were typically the most stable while complete linkage HC was consistently one of the most unstable algorithms. In summary, we found the stability to be very poor for many methods. Considering that we only removed one-fifth of data points, most methods could not achieve Jaccard similarity of $0.8$ with the remaining data points. This means that low stability approaches would be expected to perform poorly when validating results on external data sets.

**Table 3:** Mean of clustering stability of BRCA clusters for the best clustering algorithm for each different embedding based on the average of the sum of SS, clustering stability, batch effect, module and clinical relevance score across different number of clusters (k)

Dimensionality reduction^*^						Dimensionality reduction					
Embedding	Clustering	k = 3	k = 4	k = 5	k = 6	Embedding	Clustering	k = 3	k = 4	k = 5	k = 6
PCA	k-means	0.93	0.88	0.84	0.77	PCA	k-means	0.95	0.79	0.64	0.86
t-SNE	k-means	0.53	0.62	0.53	0.51	t-SNE	k-means	0.70	0.65	0.58	0.51
UMAP	k-means	0.80	0.79	0.66	0.61	UMAP	k-means	0.86	0.78	0.73	0.76
VAE	k-means	0.73	0.58	0.64	0.53	–	–	–	–	–	
DiffRank						GSVA					
Embedd.	Clustering	k = 3	k = 4	k = 5	k = 6	Embedding	Clustering	k = 3	k = 4	k = 5	k = 6
GO	DIANA	0.99	0.71	0.76	0.85	GO	HC (avg.)	0.76	0.76	0.77	0.77
KEGG	DIANA	0.91	0.84	0.82	0.70	KEGG	DIANA	0.65	0.80	0.75	0.73
Reactome	DIANA	0.97	0.89	0.92	0.93	Reactome	DIANA	0.77	0.81	0.74	0.70
Hallmark	DIANA	0.95	0.92	0.85	0.88	Hallmark	DIANA	0.67	0.79	0.72	0.65
GCN RWR-FGSEA						PPI RWR-FGSEA					
Embedding	Clustering	k = 3	k = 4	k = 5	k = 6	Embedding	Clustering	k=3	k=4	k=5	k=6
GO	DIANA	0.92	0.74	0.74	0.72	GO	DIANA	0.99	0.86	0.83	0.83
KEGG	DIANA	1.00	0.84	0.84	0.79	KEGG	HC (avg.)	1.00	0.98	0.98	0.97
Reactome	DIANA	0.82	0.55	0.74	0.72	Reactome	DIANA	0.97	0.91	0.90	0.82
Hallmark	DIANA	0.95	0.98	0.96	0.94	Hallmark	DIANA	0.95	0.93	0.94	0.91

$^{\ast }$ Applied on whole transcriptome (other results were obtained with DEG and disease associated gene intersection).

**Table 4:** Mean of clustering stability of PRAD clusters for the best clustering algorithm for each different embedding based on the average of the sum of SS, clustering stability, batch effect, module and clinical relevance score across different number of clusters (k)

Dimensionality reduction^*^						Dimensionality reduction					
Embedding	Clustering	k = 3	k = 4	k = 5	k = 6	Embedding	Clustering	k = 3	k = 4	k = 5	k = 6
PCA	k-means	0.88	0.82	0.66	0.84	PCA	k-means	0.86	0.77	0.80	0.58
t-SNE	k-means	0.45	0.37	0.34	0.34	t-SNE	k-means	0.53	0.43	0.52	0.49
UMAP	k-means	0.47	0.52	0.53	0.52	UMAP	k-means	0.73	0.58	0.65	0.56
VAE	HC (avg.)	0.86	0.82	0.78	0.76	–	–	–	–	–	
DiffRank						GSVA					
Embedd.	Clustering	k = 3	k = 4	k = 5	k = 6	Embedding	Clustering	k = 3	k = 4	k = 5	k = 6
GO	HC (avg.)	0.97	0.98	0.98	0.97	GO	DIANA	0.79	0.81	0.71	0.59
KEGG	DIANA	0.85	0.83	0.82	0.79	KEGG	HC (avg.)	0.72	0.66	0.60	0.61
Reactome	DIANA	0.63	0.51	0.53	0.52	Reactome	DIANA	0.69	0.76	0.65	0.56
Hallmark	DIANA	0.66	0.65	0.58	0.55	Hallmark	DIANA	0.71	0.68	0.61	0.55
GCN RWR-FGSEA						PPI RWR-FGSEA					
Embedding	Clustering	k = 3	k = 4	k = 5	k = 6	Embedding	Clustering	k = 3	k = 4	k = 5	k = 6
GO	DIANA	0.86	0.96	0.87	0.83	GO	DIANA	0.89	0.98	0.96	0.94
KEGG	DIANA	0.89	0.88	0.86	0.73	KEGG	HC (avg.)	0.99	0.99	0.99	0.99
Reactome	DIANA	0.89	0.87	0.89	0.84	Reactome	HC (avg.)	0.99	0.99	0.98	0.96
Hallmark	HC (avg.)	0.99	1.00	0.98	0.99	Hallmark	HC (avg.)	0.99	0.96	0.89	0.83

$^{\ast }$ Applied on whole transcriptome (other results were obtained with DEG and disease associated gene intersection).

### Evaluation of external metrics

**Figure 2:**
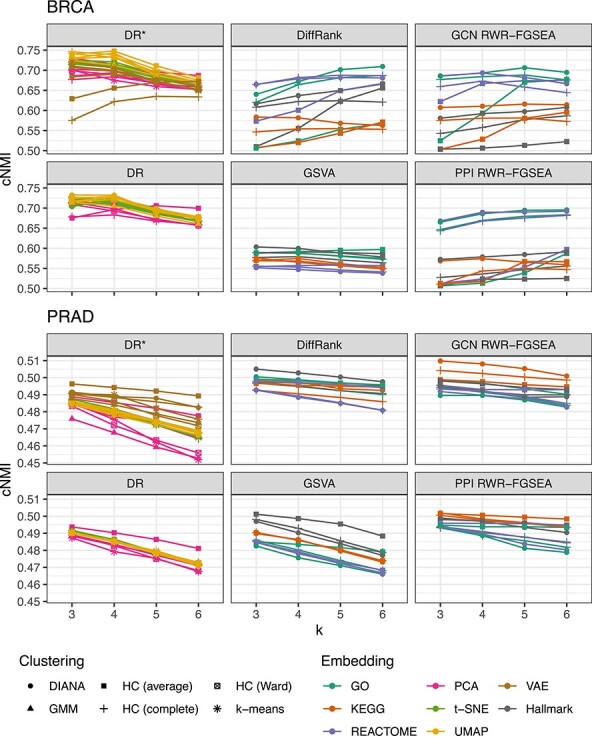
Average cNMI for different feature transformations (Embeddings), clustering algorithms and number of clusters (k) in TCGA BRCA and PRAD (PAM50 subtype NMI vs. TSS NMI and Gleason category NMI vs. TSS NMI, respectively). All values are averages from 100 resampled data subsets. While the other were obtained with the intersection of DEGs and known associations, DR^*^ corresponds to dimensionality reduction that was applied on all genes, i.e. without feature selection.

The clustering results were evaluated based on external information of patient samples (e.g. NMI between clusters and known subtypes). Moreover, we measured how robust the grouping results were with respect to know batch effects by compiling the cNMI score [47]. Figure 2 shows cNMI results for both BRCA and PRAD data sets. The constituent NMIs of the metric are shown in Supplementary Figures S6 and S7. Computational feature reduction methods seemed to slightly outperform pathway-based methods in this metric; although, some of the pathway-based approaches (e.g. DiffRank and RWR-FGSEA) outperformed DR-CL approaches in PRAD. In BRCA, batch NMI was very low compared with subtype NMI for all methods and as a result the plot corresponds mostly to subtype concordance. Since the concordance with known subtypes is lower in BK-CL methods, they could be used to highlight new subtypes in cancer diseases [7]. As expected, in BRCA, the best cNMI values in TCGA-BRCA were obtained when selecting number of clusters close to four, corresponding to Luminal A, Luminal B, Her2-enriched and Basal-like subtypes. When the number of clusters was set higher than four, the cNMI compiled over the known subtypes and batches decreases in DR-CL methods while slightly increasing in BK-CL methods. This result, together with high stability, may suggest that pathway-driven subtyping has the tendency to identify finer subdivisions. Recent studies show that breast cancer is a very heterogeneous disease and that more than four subtypes can be identified by analyzing multi-omics data [7]. Another subtype concordance metric that is commonly reported is the ARI which is shown in Supplementary Figure S8. Our subtype ARI aligned with other omics-based clustering results based on TCGA BRCA RNA-Seq data [11]. We also quantified batch effects related to the plate ID for which NMI is shown in Supplementary Figure S9. Based on our results, batch effect seems to be very low within individual TCGA cancer RNA-Seq data sets. TCGA Batch Effects Viewer [1] seems to confirm this.

### Mechanistic relevance of patient stratification results

**Figure 3:**
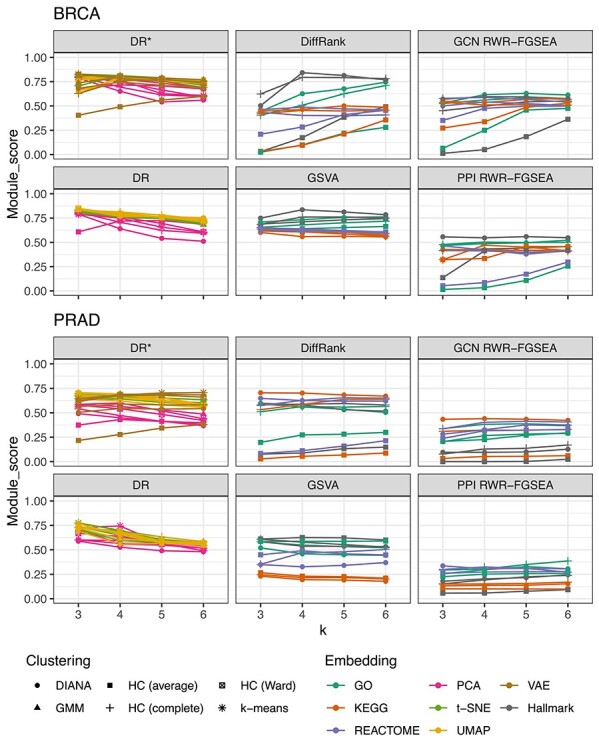
Summaries of module scores based on association with WGCNA modules as defined in Materials and Methods for different feature transformations (Embeddings), clustering algorithms and number of clusters (k) in TCGA BRCA and PRAD. All values are averages from 100 resampled data subsets. While the other were obtained with the intersection of DEGs and known associations, DR^*^ corresponds to dimensionality reduction that was applied on all genes, i.e. without feature selection.

We utilized co-expression driven gene modules to evaluate the mechanistic relevance of patient clusters. Gene modules were identified by using the WGCNA method and were used to assess whether the patient subgroups were associated with distinct disease-relevant gene modules. Details on the gene module detection are reported in Gene module score. Figure 3 shows the module scores for BRCA and PRAD. The higher the score, the more specific the disease-relevant gene modules were to patient clusters. The score measures how well all modules were associated with at least one cluster while penalizing associations with multiple clusters. DR-CL methods outperformed BK-CL methods, with some exceptions such as DiffRank in BRCA with the Hallmark gene set. Surprisingly, the network-based BK-CL methods were often outperformed by other methods. BK-CL methods utilizing KEGG pathways also tended to underperform. Network-driven approaches performed poorly in this metric, especially in PRAD where they were more likely to score $0.0$. This may be due to the fact that the variation in RWR starting points, i.e. gene seeds is much lower than variation in the gene-expression data that they were selected from. The modules were defined from correlations in DEGs while the seeds were selected from disease-associated DEGs which were a tiny subset of the module genes. Hence, it could be less probable that network-derived clusters would be associated with many different modules since the modules are topologically clustered in the network. We further analyzed the genes in each disease-relevant module with GSEA and Supplementary Figures S10–S15 show the enrichment results.

### Clinical relevance of patient stratification results

**Figure 4:**
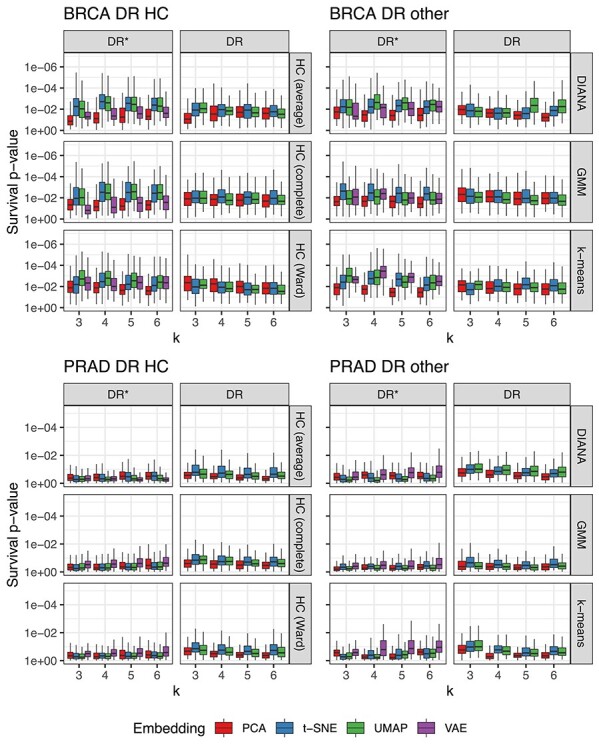
Cox-regression survival LRT $P$-value of clusters for different pathway enrichment approaches, gene set collections, clustering algorithms and number of clusters (k) in TCGA BRCA and PRAD. The plot is log-scaled and reversed such that lower $P$-values are on the top side. The box shows the 25th, 50th and 75th percentiles, while the whiskers extend from the box to $1.5\times $ interquartile range or the most extreme value depending on which is closest to the 25thor 75th percentile.

**Figure 5:**
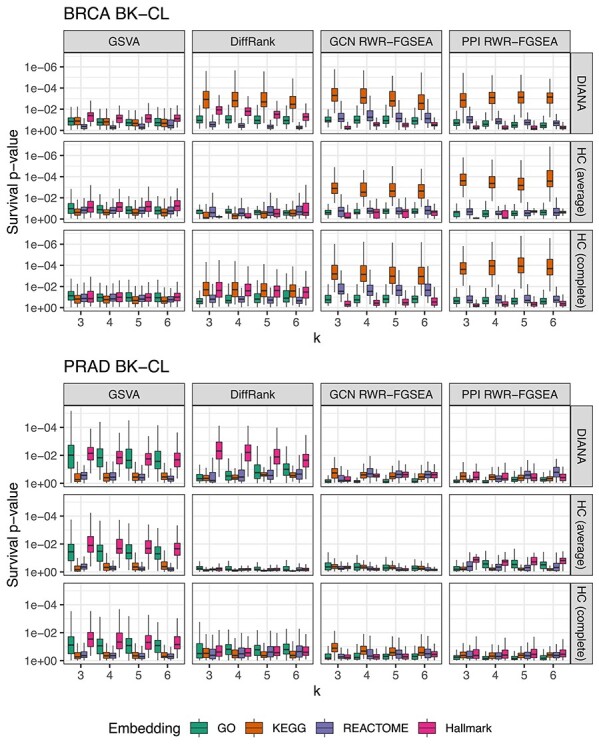
Cox-regression survival LRT $P$-value of clusters for different dimensionality reduction techniques, clustering algorithms and number of clusters (k) in TCGA BRCA and PRAD. The plot is log-scaled and reversed such that lower $P$-values are on the top side. The box shows the 25th, 50th and 75th percentiles, while the whiskers extend from the box to $1.5\times $ interquartile range or the most extreme value depending on which is closest to the 25th or 75thpercentile. DR results were obtained with the intersection of DEGs and known associations, while DR^*^ corresponds to dimensionality reduction that was applied on all genes, i.e. without feature selection.

To evaluate the survival relevance of clusters we compared Cox-regression model LRT $P$-values. A reference model based on clinical covariates was compared with models with additional cluster indicator variables. For BRCA, the selected survival model covariates were age at diagnosis and tumor stage while for PRAD pre-operative PSA and tumor N-stage were used. The LRT compares the likelihoods of two competing models and it accounts for different degrees of freedom (i.e. number of clusters) between them. Figures 4 and 5 show the distribution of $P$-values of survival differences between clustering results for the resampled data sets. DiffRank and RWR-FGSEA yielded significant survival differences when paired with KEGG pathways in BRCA, while other BK-CL methods yielded mostly insignificant results. However, in PRAD, the results were different: GSVA with GO-BP pathways performed well when other BK-CL methods did not. Interestingly, the Hallmark gene sets did not yield high survival relevance in BRCA but in PRAD they were as relevant as GO-BP. However, further analysis revealed these results to be of low stability, while, in contrast, the GO-BP clusters were relatively stable. The choice of clustering method was relevant with DIANA yielding the most significant differences. In BRCA DR-CL methods were often more significant than most BK-CL methods. Also, the highest median significance in BRCA survival was observed between four clusters derived from VAE embeddings with k-means. Nevertheless, the best BK-CL approaches outperformed DR-CL approaches most of the time. In PRAD, DR-CL did not yield any significant survival differences.

One possible explanation for the differences in the survival relevance of the clusters between BRCA and PRAD could be the set of disease-associated genes. As mentioned in Materials and Methods, BRCA-associated genes were selected based on a genetic association score derived from GWAS-based evidence. In contrast, in the Open Targets database the PRAD-associated genes were selected based on the overall score which is derived from several sources. The quality of the set of disease-associated genes could be different; although, based on the lack of significant differences between DR-CL results using different gene filters in both data sets, this might not be the case. In fact, by using the whole transcriptome as input to DR-CL in BRCA slightly better $P$-values were obtained with t-SNE and UMAP. The number of samples in the data sets also directly affects the $P$-value of the LRT, which could explain why PRAD results were poorer overall. The difference between BK-CL approaches could stem from the fact that GSVA and DiffRank are perhaps more sensitive to smaller changes in gene-expression of a few genes due to how they compute gene statistics relative to other samples.

It is currently known that predicting an individual patients’ risk of recurrence or metastatic progression by using gene expression data in PRAD remains challenging [27]. These results suggest that BK-CL approaches can obtain clinically more relevant patient subgroups. Combining known disease-gene associations and pathway enrichment yielded significant differences in survival between clusters. In contrast, using the disease-associated genes in the data-driven clustering approaches did not yield significant improvements over using all available genes as input features. While the BK-CL approaches performed well on these data sets under the given conditions, it could be argued that they benefited from the fact that both breast cancer and prostate cancer are already well studied and, as such, the pathways and disease-associated genes were able to highlight relevant aspects of the data. Indeed, the survival significance of the pathway-enrichment-based BRCA clusters tended to be higher than the known subtypes which had a LRT $P$-value of $0.0056$ on this data when tested in a similar way to the clusters. Based on the metrics of our network diffusion approach, the gene co-expression network that we inferred from the tumor expression data performed similarly to the PPI network. Gene-expression is not always correlated with the corresponding protein concentrations [18], as recently shown for both BRCA and PRAD [36, 48]. Thus, perhaps the true molecular interactions are not captured by our method but the genes that are loosely related to multiple affected genes are discoverable with our method and point to pathways relevant to survival. We also tested the use of RWR affinities as inputs to the DR-CL approach, but the results were roughly equivalent to the DR-CL results suggesting that the combination of networks and pathways is beneficial. Another benefit of the BK-CL approaches for determining clinical relevance is that the results are readily interpretable.

### RWR-FGSEA based breast cancer clusters

**Figure 6:**
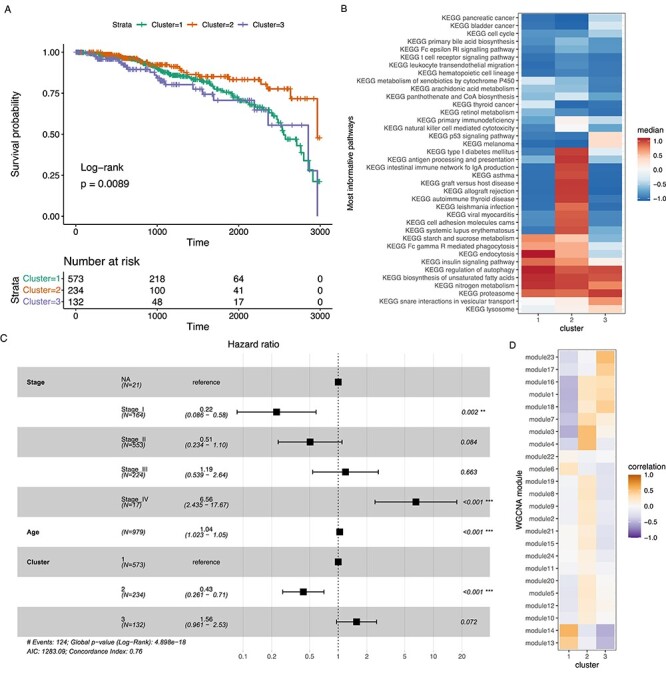
TCGA BRCA clusters from GCN RWR-FGSEA and DIANA. Panel A shows a Kaplan–Meier survival plot for the clusters, B shows root mean square scaled medians of the most informative pathway activity scores, C shows the hazard ratios of the Cox PH model including covariates, D shows correlations between clusters and the WGCNA module eigen-genes.

We selected one of the best clustering results for BRCA to analyze further, by using the first Pareto frontier plot shown in Supplementary Figure S16. The selection process is described in more detail in the Supplementary Methods and Results file. Figure 6A–D shows results of this best patient stratification involving three clusters. Survival analysis of BRCA patients belonging to the identified clusters (Figure 6A) shows that, compared with the most common first cluster with average prognosis, the second cluster had a more positive long-term prognosis while the third cluster was inseparable. Figure 6B shows medians of scaled RWR-FGSEA activity scores for pathways exhibiting the largest differences between clusters as identified by a BH-adjusted Kruskal–Wallis test $P<{10}^{-50}$. Positive and negative RWR-FGSEA pathway activity scores are associated with different RWR seed genes. Positive scores are associated with DEGs with positive FC while negative scores are associated with DEGs with negative FC. The results indicate modulation of several cancer, cell cycle and metabolism-related pathways that are shared between all the clusters. Clusters 1 and 3 differ mostly on vesicular transport-related pathways modulating endocytosis, phagocytosis and lysosomal functions. Interestingly, the separation of cluster two results mostly on modulation of several immune-related pathways indicating higher expression and activity of immunomodulatory functions in tumors of this patient group with better survival. Expression of genes in the most informative pathways is shown in Supplementary Figure S18. Figure 6C shows the hazard ratios of a Cox PH model based on tumor stage, patient age and the identified clusters. Supporting the results from the survival analysis, the second cluster was associated with a significantly lower risk while the third cluster was associated with higher risk when compared with the first cluster. Supplementary Table S6 shows the clinical variable distribution within the clusters. Figure 6D shows Spearman correlation between clusters and disease module eigen-genes which were identified by using WGCNA. Overall, module correlation with specific modules was not very high, which aligns with the mediocre module score observed in the Pareto front (Supplementary Figure S16). Module to pathway associations based on GSEA for breast cancer are shown in Supplementary Figures S10–S12.

### GSVA-based prostate cancer clusters

**Figure 7:**
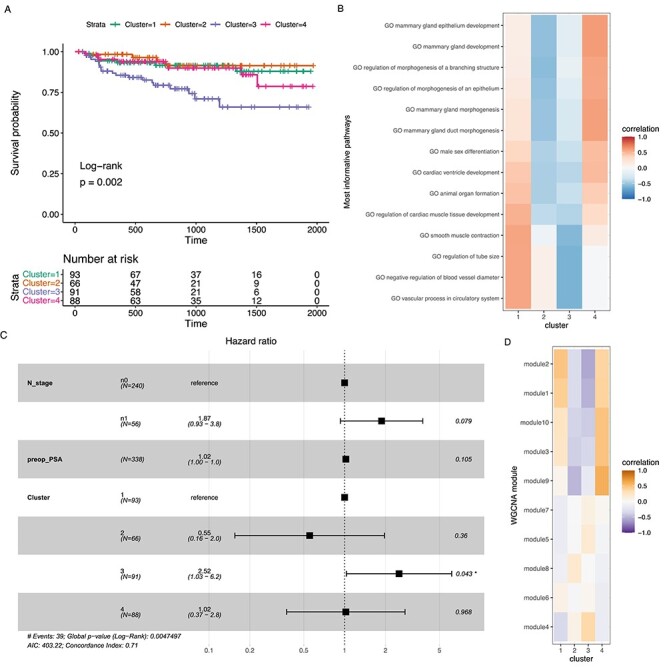
TCGA PRAD clusters from GSVA and DIANA. Panel A shows a Kaplan–Meier survival plot for the clusters, B shows correlations between cluster indicators and the most informative pathway activity scores, C shows the hazard ratios of the Cox PH model including covariates, D shows correlations between clusters and the WGCNA module eigen-genes.

We also selected the best clustering result for PRAD to analyze further, similarly to BRCA, by selecting one of the Pareto optimal solutions (Supplementary Figure S18). Figure 7A–D shows results of the best method dividing the patients into four clusters. Survival analysis of PRAD patients corresponding to the selected clusters (Figure 7A) indicates three clusters with insignificant differences in biochemical recurrence, and one cluster with significantly poorer outcome. Figure 7B shows Spearman correlation between the clusters and GSVA enriched pathways exhibiting the largest differences between clusters as identified by a BH-adjusted Kruskal–Wallis test $P<{10}^{-50}$. GSVA scores pathways based on differences in gene expression relative to other tumor samples within each pathway, a higher score corresponds to overall higher relative expression and vice versa. Cluster three with poor outcome has implications of decreased level of epithelial differentiation, supportive of the idea of more aggressive tumors. Interestingly, also cluster 2, with a trend to best survival among the clusters, also shares this downregulation of epithelial differentiation pathways while both clusters 1 and 4 have increased activity scores on these, supporting the idea of well-differentiated, less aggressive tumors. Cluster 3 is distinguished by downregulation of smooth muscle components and pathways on vascular functions. While the first may indicate decreased amount of stroma in more aggressive tumors, both may indicate functions related to providing enhanced circulation and less dense supportive structures supporting faster growth and abilities to metastasize. Expression of genes in the most informative pathways is shown in Supplementary Figure S19. Figure 7C shows the hazard ratios of a Cox PH model based on tumor N-stage, pre-operative PSA and cluster, verifying that the identified cluster number three had a higher risk of biochemical recurrence. Supplementary Table S7 shows the clinical variable distribution within the clusters. Figure 7D shows correlation between clusters and disease module eigen-genes generated with WGCNA. Supplementary Figures S13–S15 show GSEA based enrichment analysis results for the modules.

## Discussion

In this study, we aimed to compare data-driven with knowledge-driven methods for patient stratification from cancer gene expression data. Widely used dimensionality reduction techniques, such as PCA, t-SNE, UMAP and VAEs, were compared against methods that reduce gene-level information into pathway activation scores over a sample population. Both strategies were coupled with standard clustering algorithms, such as agglomerative or divisive HC, Gaussian mixture models and k-means. Metric fairness and usefulness are limitations which we attempted to address by introducing the module score and survival-based metrics. Different evaluation metrics were used to assess their performance including several internal and external validation measures, as well as scores aiming to evaluate the clinical utility and the mechanistic relevance of the identified patient groupings. When using the DR-CL approach, the choice of DR method had more impact than the choice of clustering algorithm. In terms of common clustering metrics, such as cluster separability, cohesion and gold-standard agreement, manifold learning methods, especially UMAP, outperformed PCA. This might be attributed to the manifolds’ ability to preserve local as well as global structure in the data [3]. However, in terms of clustering stability, PCA performed well while UMAP and t-SNE had poor performance. In general, t-SNE and UMAP are not recommended for clustering since their primary intended use is data visualization and the distance between data points in the embedded space is not preserved accurately. Nevertheless, the use of t-SNE in combination with clustering algorithms has been common in the field of bioinformatics, even though poor stability of the result might be expected. Our analysis showed that UMAP-based clustering consistently achieved higher stability in comparison to t-SNE in these two data sets.

The significance of the embedding was also apparent in the BK-CL results: the gene set collection choice was more significant than the clustering algorithm in every metric. This suggests that studying different DR and BK integration methods is relevant. BK-CL methods were outperformed by DR-CL methods when considering SS and the gene modulation score. With some exceptions, BK-CL had better results in stability and clinical relevance of the compiled subtypes. Regarding the cNMI scores, DiffRank and RWR-FGSEA methods outperformed DR-CL methods in PRAD. In BRCA it was interesting to observe that the cNMI score compiled for DiffRank-based solutions increases when considering a higher number of clusters, suggesting that pathway-driven subtyping may help the identification of finer subdivisions in known subtypes. In both BRCA and PRAD, the best clustering results did not adhere to the current clinical divisions and could therefore provide additional value. It should be noted that differences in treatment were ignored; hence, the differences could be related to differences in efficacy of treatments for patients in the clusters.

In summary, no single approach dominated every metric, showing the importance of multi-objective evaluation of clustering results. Out of the five metrics, cNMI and survival analysis were perhaps most relevant since the SS, clustering stability and module score were likely to be more affected by technical choices in the methods rather than any biologically linked reasons. Pathway-based approaches are very promising and many different methods have been suggested [66]. Although systematic comparison between them is complicated due to excessive computational time requirements and inadequate automation for systematic clustering analysis. In the future, it would be interesting to use sub-pathways and analysis of signal transduction within directed circuits in pathways, which could provide more meaningful functional activity estimation and, thus, better patient-specific pathway profiles [20]. Highly scalable pathway enrichment methods, such as DiffRank, could also be applied to cluster scRNA-Seq data.

**Key Points**

-
$\bullet $ A systematic comparison of dimensionality reduction and biological knowledge (BK) integration methods in clustering workflows is provided.
-
$\bullet $ A multi-objective evaluation procedure is provided for assessing the goodness of clustering results based on standard metrics, such as group separability and stability measures, and metrics assessing clinical and mechanistic significance of the identified subtypes.
-
$\bullet $ The benchmarking results showed that no single method dominated every metric showing the importance of multi-objective evaluation.
-
$\bullet $ BK-based clustering approaches yielded results with the highest prognostic value in both breast cancer and prostate cancer.
-
$\bullet $ We identified clusters with significant prognostic value that were distinct from current clinical division in both breast cancer and prostate cancer.

## Supplementary Material

FileS1_Supplementary_Methods_and_Results_Rev_bbab314

FileS2_BRCA_metrics_bbab314

FileS3_PRAD_metrics_bbab314

FileS4_DRCL_DEG_metrics_bbab314

## Notes

**Teemu J. Rintala** received his MSc (tech.) in life science technologies from Aalto University School of Science, Finland, in 2019. He is currently an Early-stage Researcher and PhD candidate at the Institute of Biomedicine, University of Eastern Finland, Finland. His research interests include bioinformatics and machine learning applications in precision medicine.

**Antonio Federico** is a Postdoctoral researcher at the Finnish Hub for Development and Validation of Integrated Approaches (FHAIVE) - Tampere University (Finland). He is specialized in computational analyses of high-throughput sequencing data and systems biology modelling in human diseases.

**Leena Latonen** is K.Albin Johansson Cancer Research Fellow of the Finnish Cancer Institute and leads a research group studying molecular and tissue aberrations of hormone-dependent cancers at University of Eastern Finland.

**Dario Greco** is professor of bioinformatics at Tampere University and director of FHAIVE (the Finnish Hub for Development and Validation of Integrated Approaches). His research is focused on systems toxicology and pharmacology.

**Vittorio Fortino** is academy research fellow at Academy of Finland and leader of the Biomedical Informatics (BI) research group at University of Eastern Finland. His research focus is on Artificial Intelligence and Machine Learning methods for biomarker, disease subtype and drug discoveries.
