# Supplementary material for: A systematic comparison of data- and knowledge-driven approaches to disease subtype discovery
Source: Brief Bioinform. 2021 Aug 13;22(6):bbab314. doi: 10.1093/bib/bbab314 (PMC8575038; doi:10.1093/bib/bbab314)
Supplement: FileS1_Supplementary_Methods_and_Results_Rev_bbab314 [file files1_supplementary_methods_and_results_rev_bbab314.pdf]

Supplementary Methods and Results for: A systematic comparison of data- and knowledge-driven approaches to disease subtype discovery

Teemu J. Rintala[1], Antonio Federico[2,3], Leena Latonen[1], Dario Greco[2,3,4], and Vittorio Fortino[1,*]

[1]Institute of Biomedicine, University of Eastern Finland, Yliopistonranta 1 E, 70210 Kuopio, Finland
[2]Faculty of Medicine and Health Technology, Tampere University, Kalevantie 4, 33100, Tampere, Finland
[3]BioMediTech Institute, Tampere University, Kalevantie 4, 33100, Tampere, Finland
[4]Institute of Biotechnology, University of Helsinki, Viikinkaari 5d, 00014, Helsinki, Finland
[*]Corresponding author. vittorio.fortino@uef.fi

July 5, 2021

1 Expanded methods

1.1 Variational autoencoder implementation

Variational Autoencoder is a variational-Bayes extension of an autoencoders which is an unsupervised Deep Learning method that uses multiple layers of non-linear functions to transform input data into a low dimensional representation and then aims reconstruct the original data from this embedding [1]. We used tensorflow 2.0 in Python to implement simple fully connected VAE with 7 layers of hidden units: Three dense layers with 256, 128 and 64 rectified linear units were used on the encoder side and mirrored on the decoder side. The embedding layer was set up for 10 dimensions i.e. the last encoder layer was used to generate 10 μ and 10 σ parameters corresponding to the multivariate normal distribution of the 10 dimensional embedding. No activation function was used on the embedding layer and all weights were initialized uniformly [2]. We used the Adam optimizer [3] with learning rate $\gamma = 0.0001$, moment decay parameters $\beta_1 = 0.9$, $\beta_2 = 0.999$ and stability

parameter $\epsilon = 10^{-7}$ to train the VAE. The training data was fed to the optimizer in mini-batches of 128 samples. To minimize overfitting we applied three common regularization strategies: a L2 norm penalty on the weights, dropout and stopping early when the reconstruction error on a holdout set of samples starts to increase. The L2 penalty penalizes large weights while dropout penalizes models that rely on a small set of input features by setting a random subset of all inputs to zero in each training iteration. The penalty term was scaled with $\lambda = 100$ and dropout-rate was set to 0.2. Early stopping with patience equal to 100 epochs was implemented and the model was trained for a maximum of 2000 epochs. Different hyper-parameter values for λ, γ, dropout probability and patience were first tested with a random search. The aforementioned values were selected based on the initial results. Additionally, the gene-expression values were centered for the VAE based analysis so that the dropout would effectively set the affected genes to the mean values within the sample.

1.2 Additional clustering methods

We also tested three more recent clustering methods namely the DR and consensus clustering based SC3 [4], spectral clustering of nearest neighbor graph kernels with Spectrum [5] and Louvain community detection [6] on shared nearest neighbor graphs which is equivalent to the default settings of Phenograph [7]. The results did not stand out compared to the methods in the main article but are given in Supplementary Tables S1 and S2 (on sheet 5).

1.3 Multiple-criteria decision analysis of patient stratification results

In this section we explore the trade-offs between the evaluation criteria that were implemented to compare the different patient stratification methods. To this end we adopted the concept of Pareto front or set of optimal solutions in the space of objective functions in multi-objective optimization problems. An ideal patient stratification solution would simultaneously optimize each evaluation criteria (or objective). However, often, no single solution exists that is optimal with respect to every objective because of conflicting factors. Although we cannot demonstrate whether or not the defined evaluation metrics are in conflict, we can still use the concept of Pareto-set in order to highlight clustering results that are non-dominant to each other (when using five evaluation criteria – silhouette score, cNMI, survival statistics and clustering stability) but are superior to the rest of clustering solutions. Figure S16 shows a summary of evaluation metrics compiled for clustering results in the first Pareto frontier computed with the *rPref*-package [8]. Results with low stability (< 0.8) were excluded. The survival p-values

were binned into significance categories and scored with values between 0 and 1 based on the following function:

$$f(p) = \begin{cases} 0 & \text{if } p \geq 0.05 \\ 0.25 & \text{if } 0.01 \leq p < 0.05 \\ 0.5 & \text{if } 0.001 \leq p < 0.01 \\ 1 & \text{if } p < 0.001 \end{cases}. \quad (1)$$

For TCGA BRCA there were several viable solutions, but perhaps the most interesting was the highly stable GCN RWR-FGSEA based result with three clusters which had one of the most significant survival difference and higher module score than the PPI based alternative suggesting a more comprehensive coverage of disease mechanisms. The most significant survival differences were observed for a VAE based clustering result, but it was excluded due to poor clustering stability. For TCGA PRAD, we selected the result with the highest survival difference which was based on GSVA with Gene Ontology gene sets.

2 Supplementary figures and tables

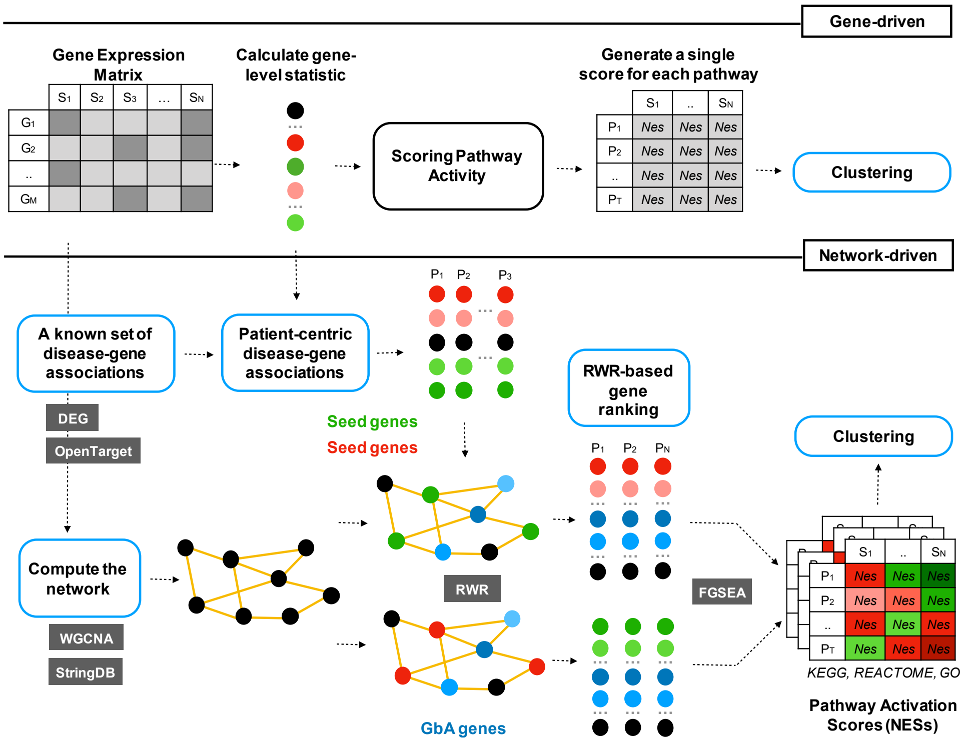

Figure S1: Pathway scoring methods such as GSVA and DiffRank use gene level statistics derived from the gene expression matrix to produce pathway activity scores for arbitrary gene sets (usually based on pathway annotations from various databases). The novel RWR based approach expands a set of patient specific gene seeds in a molecular interaction network as an additional step before enrichment analysis. This results in higher scores for pathways with affected genes that are close in the interaction network.

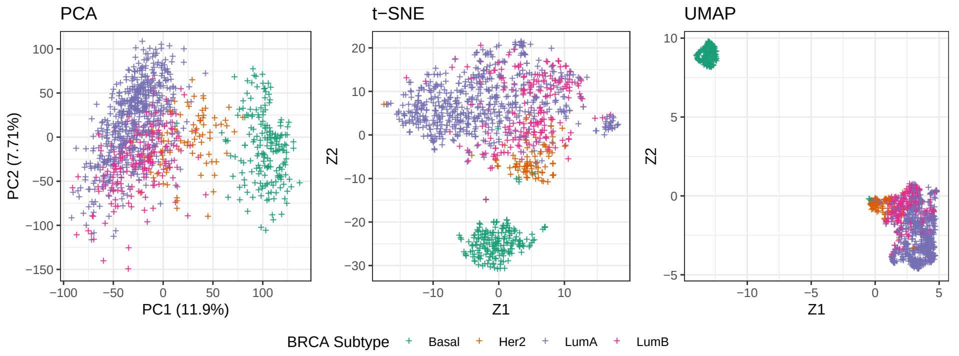

Figure S2: BRCA gene-expression data visualizations based on PCA, t-SNE and UMAP.

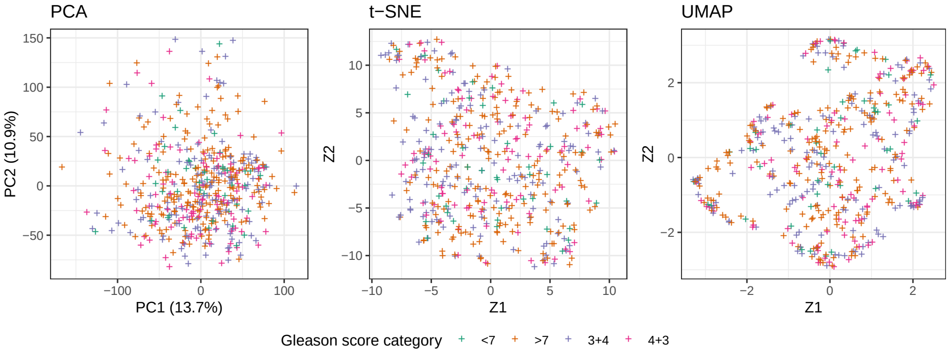

Figure S3: PRAD gene-expression data visualizations based on PCA, t-SNE and UMAP.

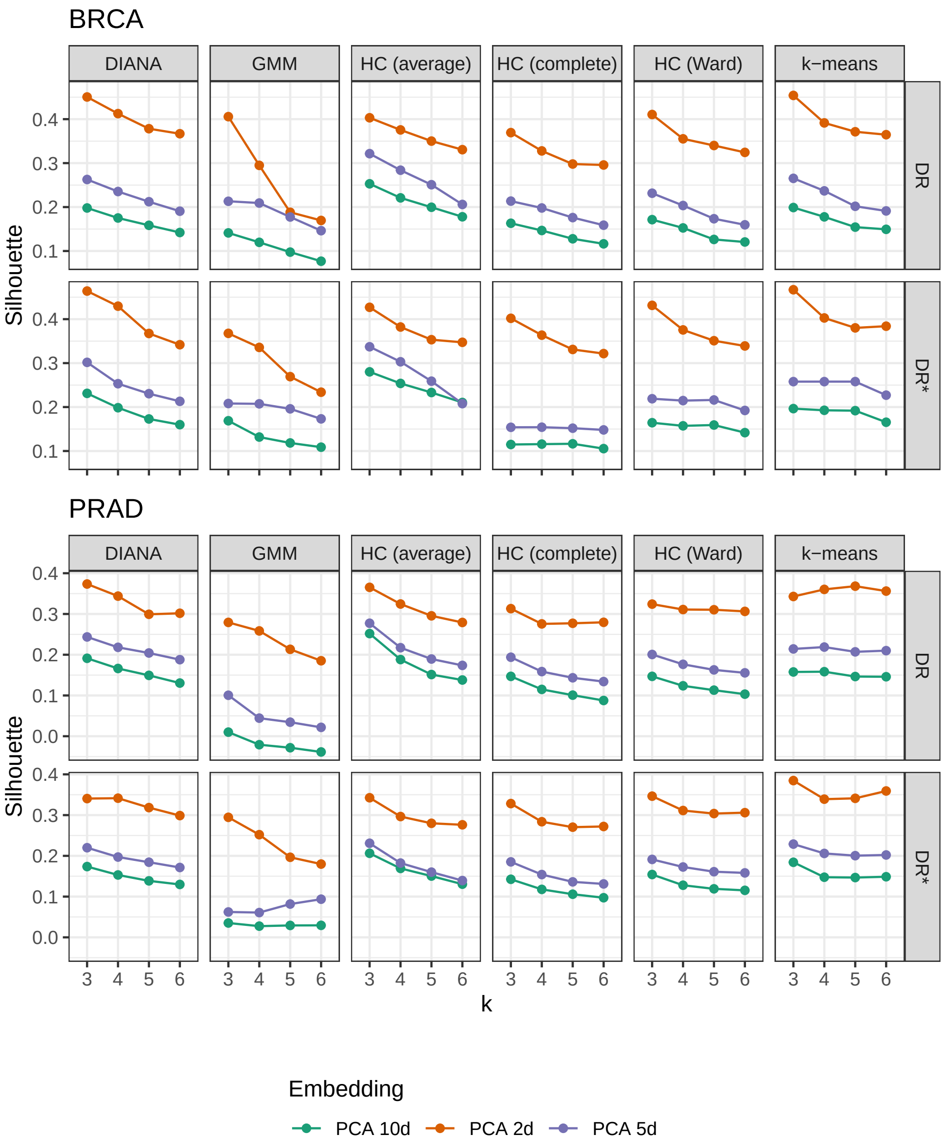

Figure S4: Silhouette score for PCA with different number of dimensions and clustering algorithms in TCGA BRCA and PRAD. All values are averages from 100 resampled data subsets. DR* corresponds to dimensionality reduction that was applied on all genes i.e. without feature selection. Silhouette score is greatly affected by the number of dimensions.

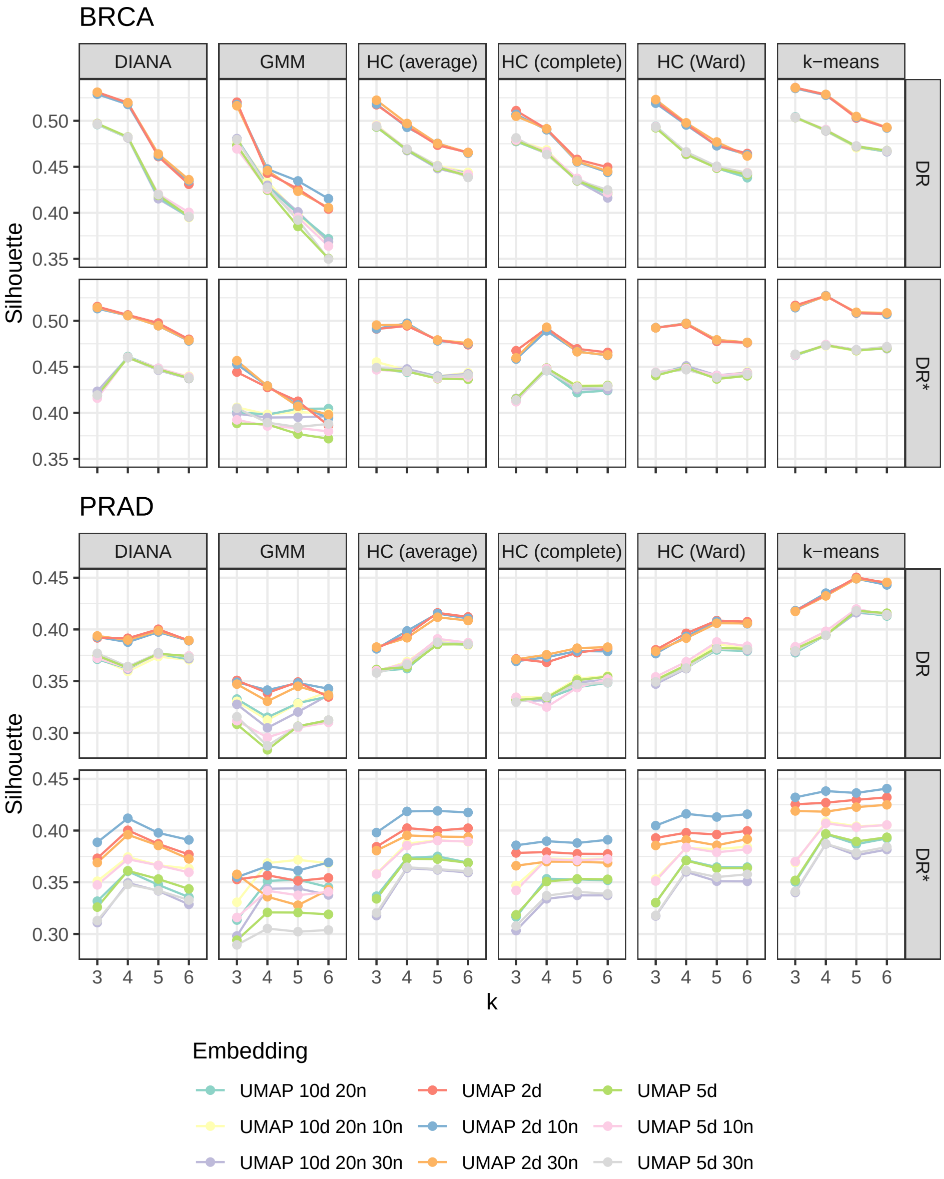

Figure S5: Silhouette score for UMAP with different number of dimensions, number of neighbors and different clustering algorithms in TCGA BRCA and PRAD. All values are averages from 100 resampled data subsets. DR* corresponds to dimensionality reduction that was applied on all genes i.e. without feature selection. Silhouette score seems to depend on the number of dimensions to an extent, but 5 and 10 dimensions yield almost identical results for most clustering algorithms.

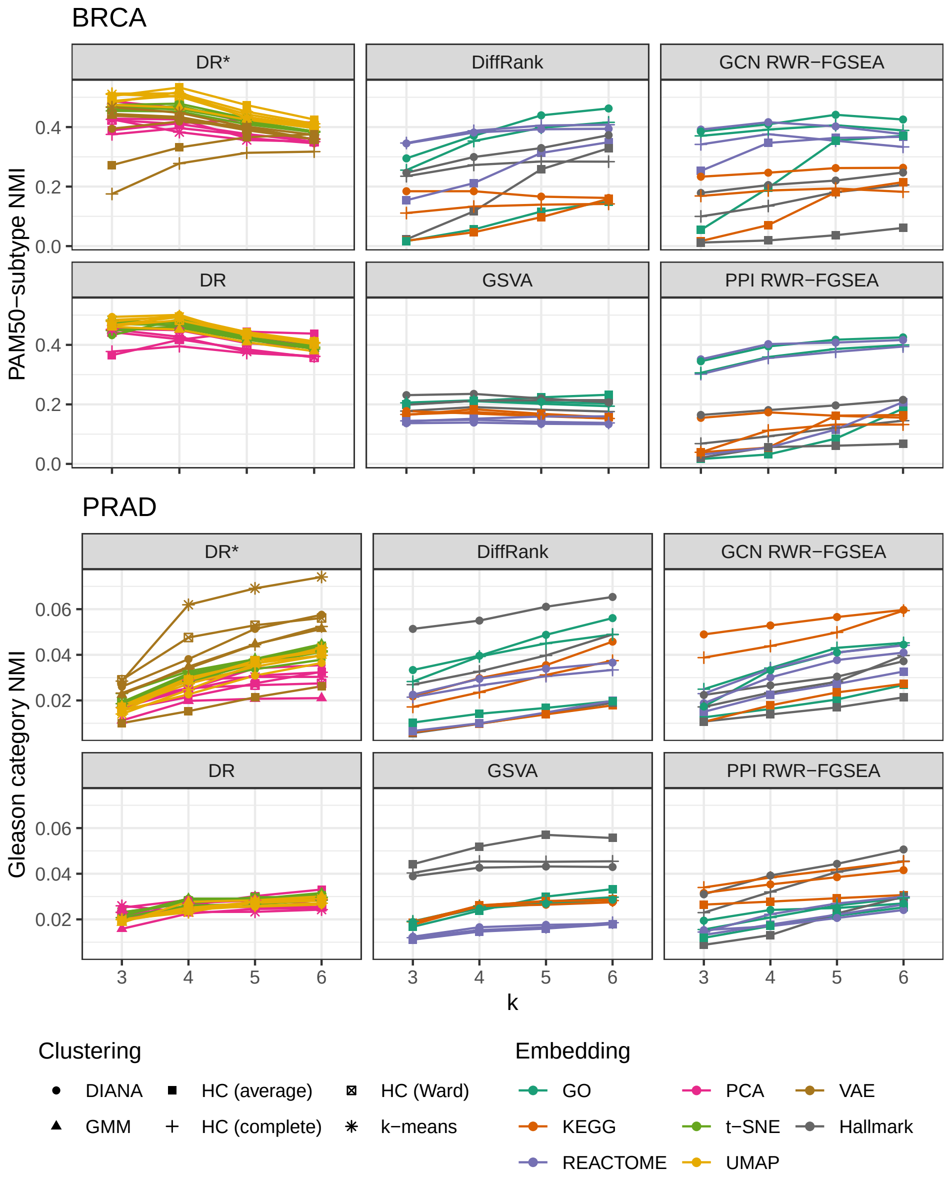

Figure S6: Subtype NMI for different feature transformations and clustering algorithms in TCGA BRCA and PRAD. All values are averages from 100 resampled data subsets. DR* corresponds to dimensionality reduction that was applied on all genes i.e. without feature selection.

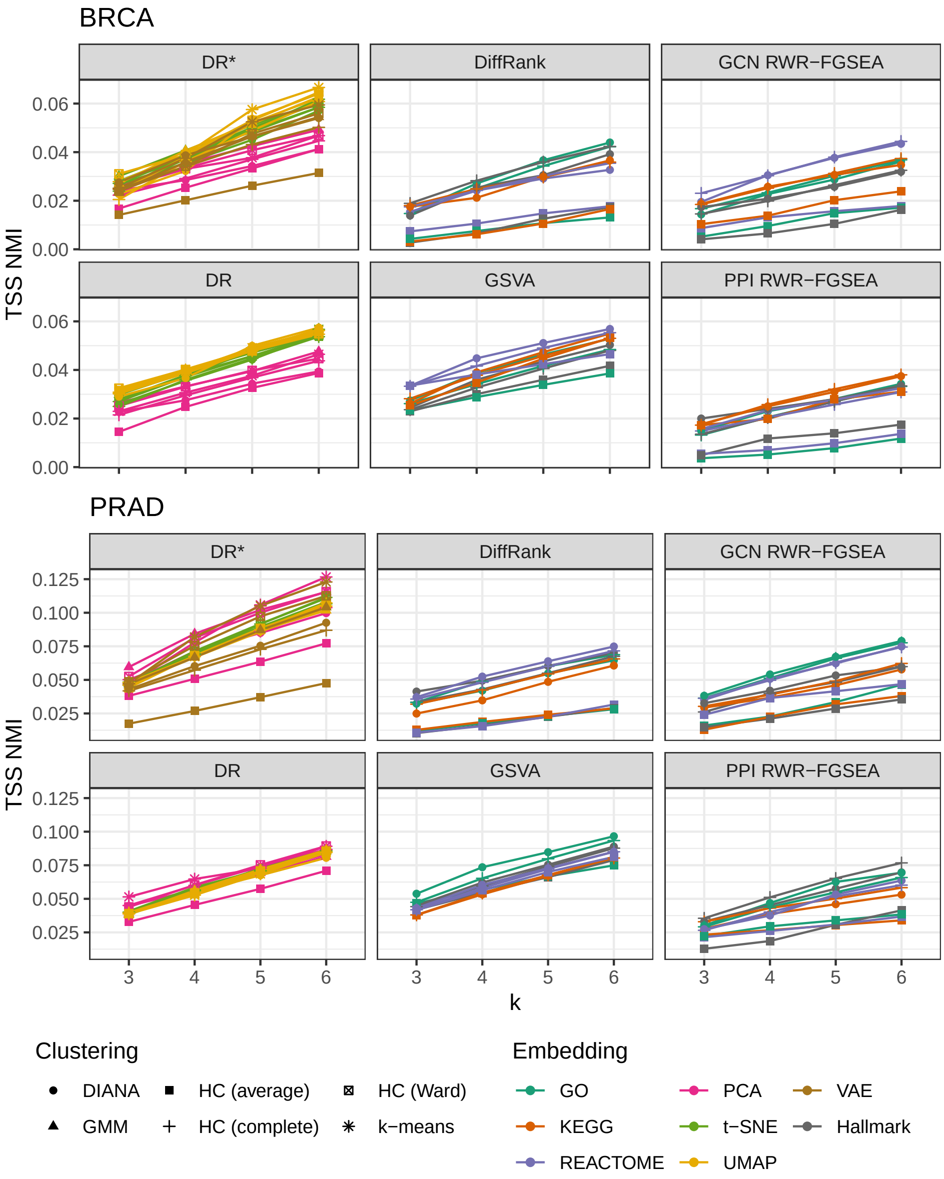

Figure S7: TSS NMI for different feature transformations and clustering algorithms in TCGA BRCA and PRAD. All values are averages from 100 resampled data subsets. DR* corresponds to dimensionality reduction that was applied on all genes i.e. without feature selection.

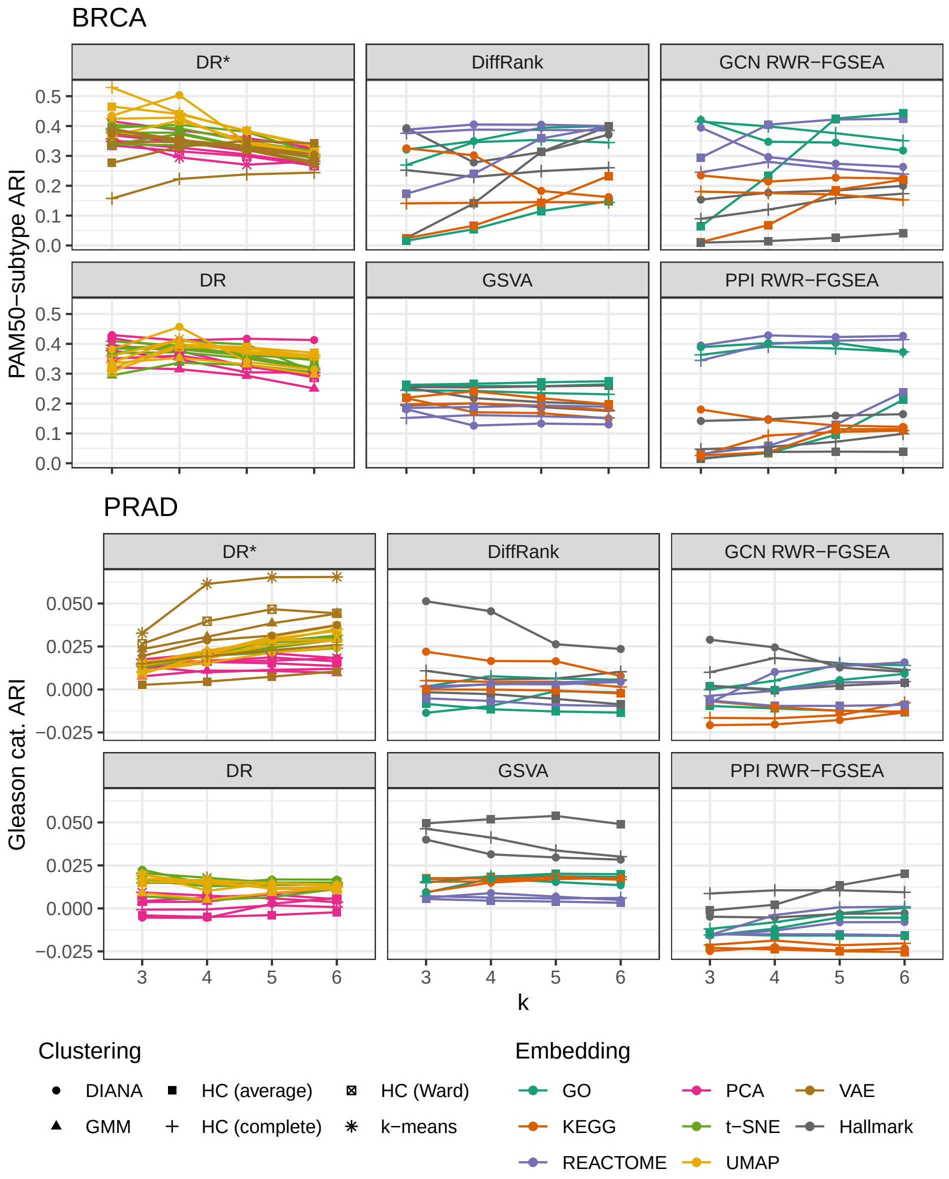

Figure S8: Subtype ARI for different feature transformations and clustering algorithms in TCGA BRCA and PRAD. All values are averages from 100 resampled data subsets. DR* corresponds to dimensionality reduction that was applied on all genes i.e. without feature selection.

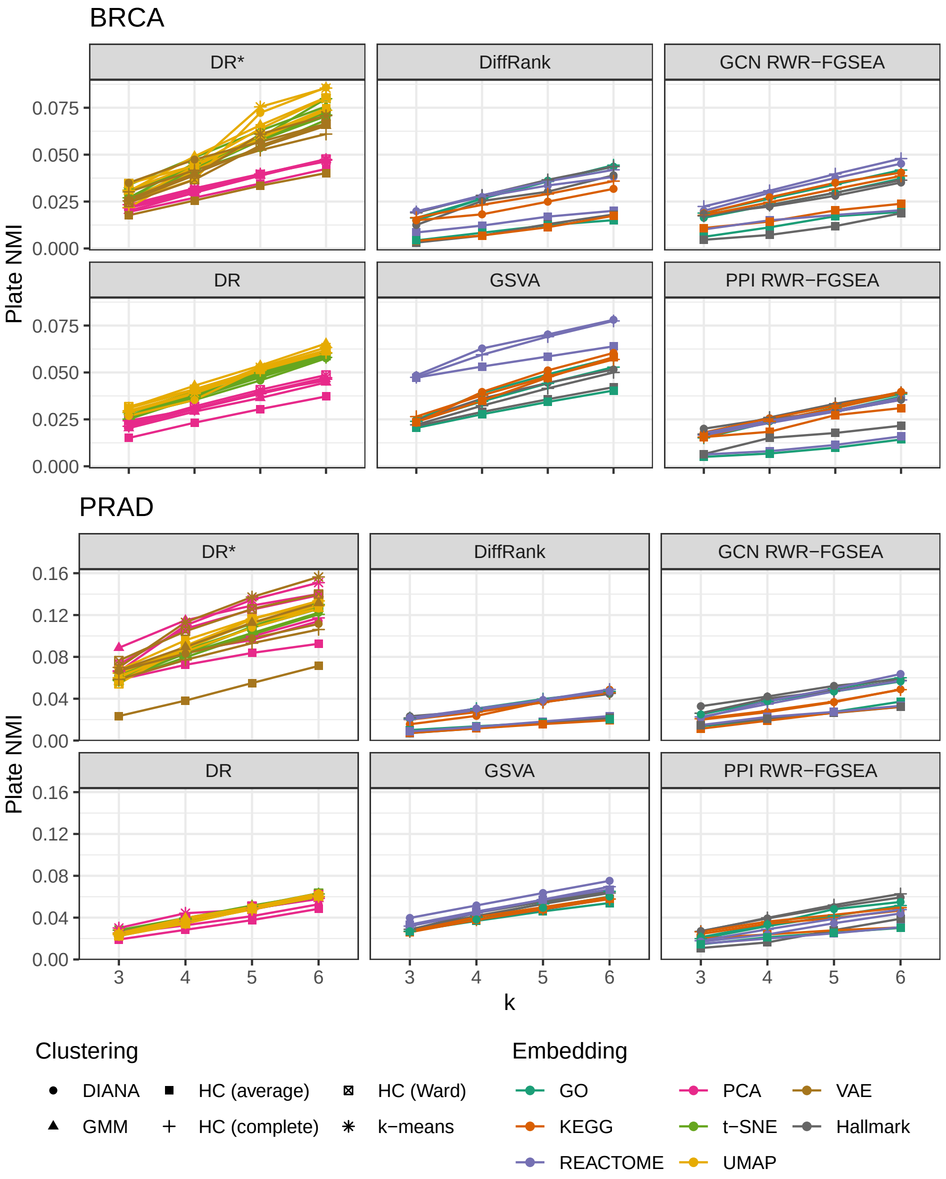

Figure S9: Plate NMI for different feature transformations and clustering algorithms in TCGA BRCA and PRAD. All values are averages from 100 resampled data subsets. DR* corresponds to dimensionality reduction that was applied on all genes i.e. without feature selection.

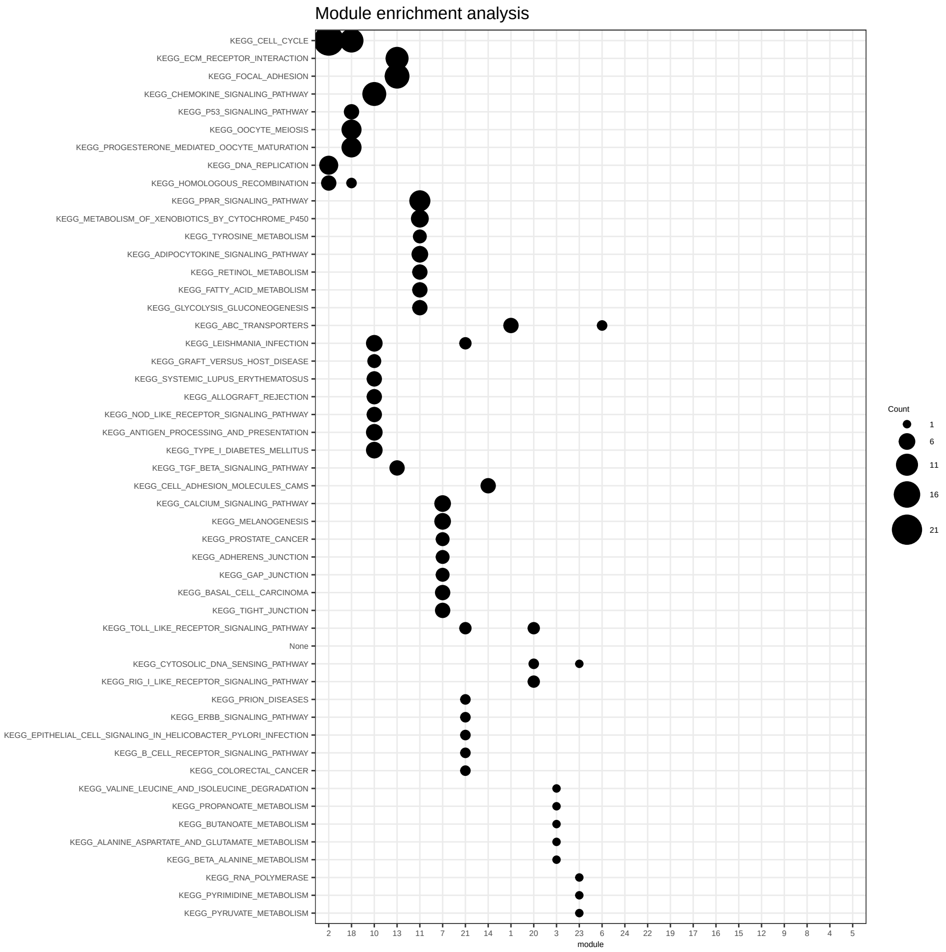

Figure S10: BRCA WGCNA module KEGG GSEA.

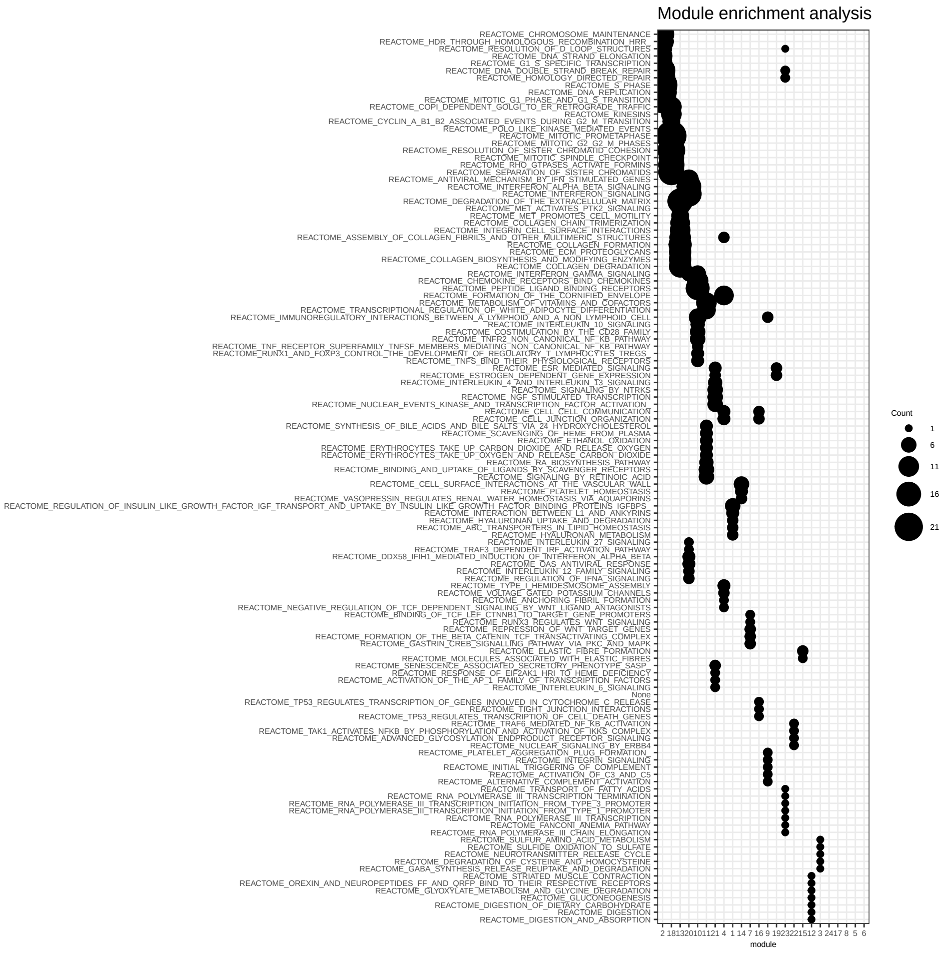

Figure S11: BRCA WGCNA module REACTOME GSEA.

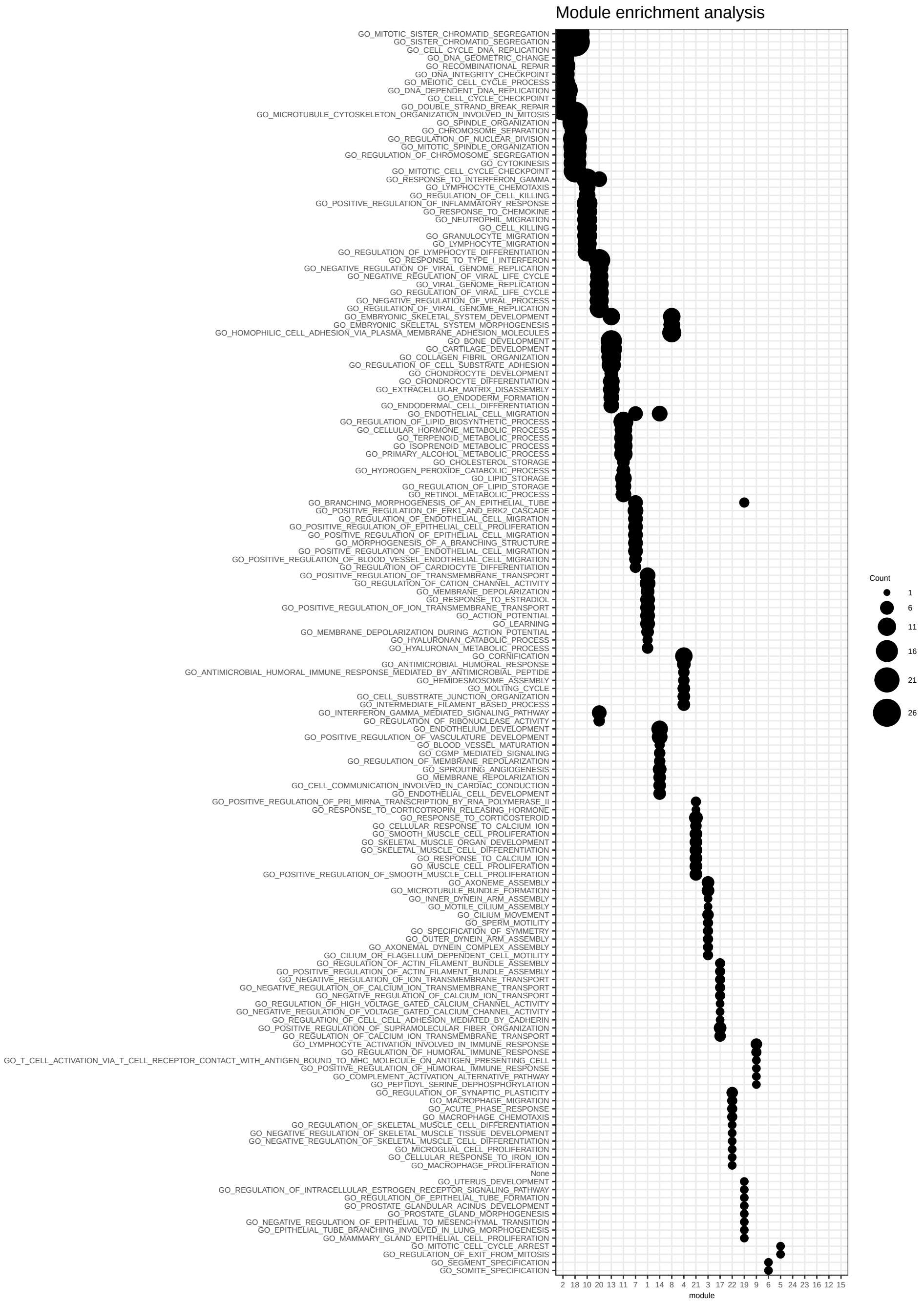

Figure S12: BRCA WGCNA module GO GSEA.

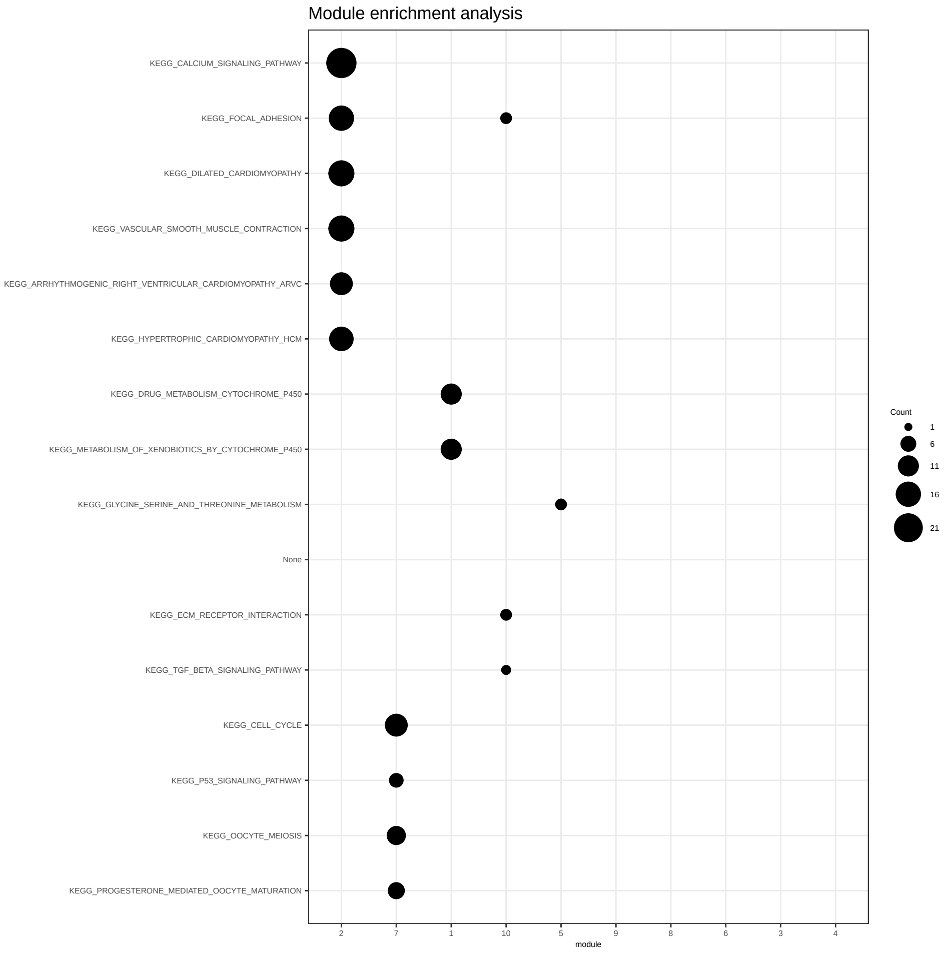

Figure S13: PRAD WGCNA module KEGG GSEA.

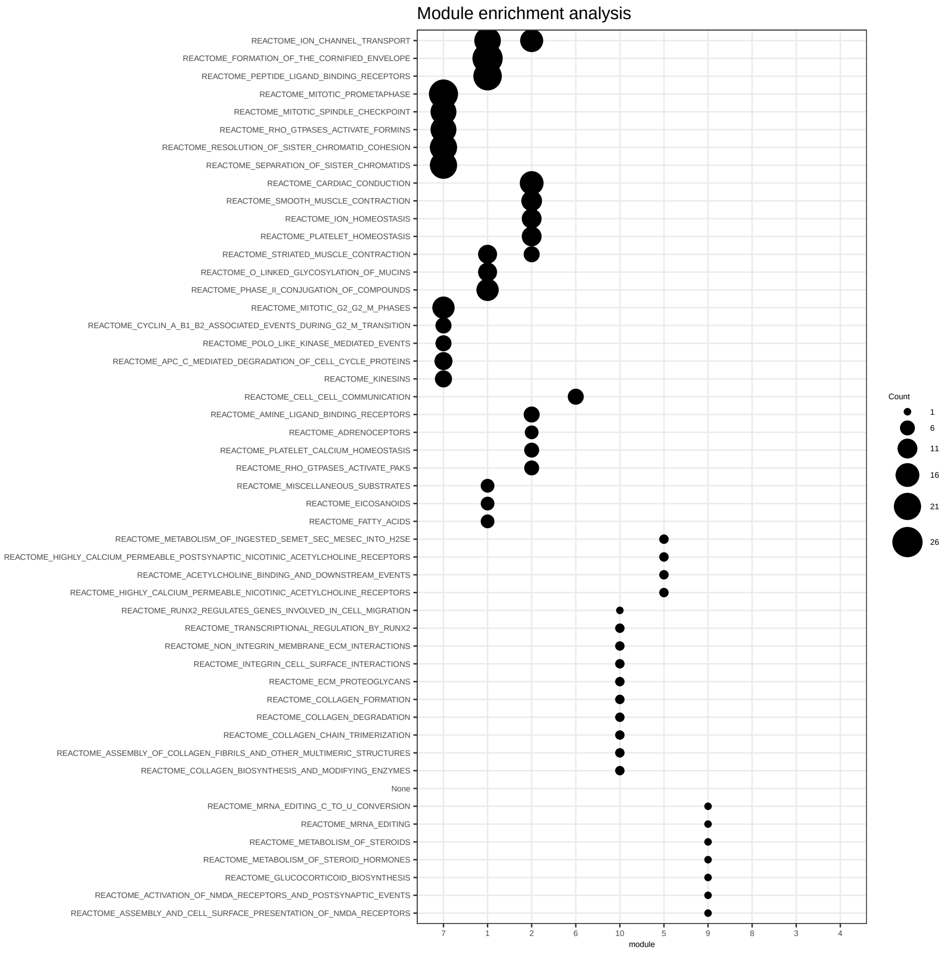

Figure S14: PRAD WGCNA module REACTOME GSEA.

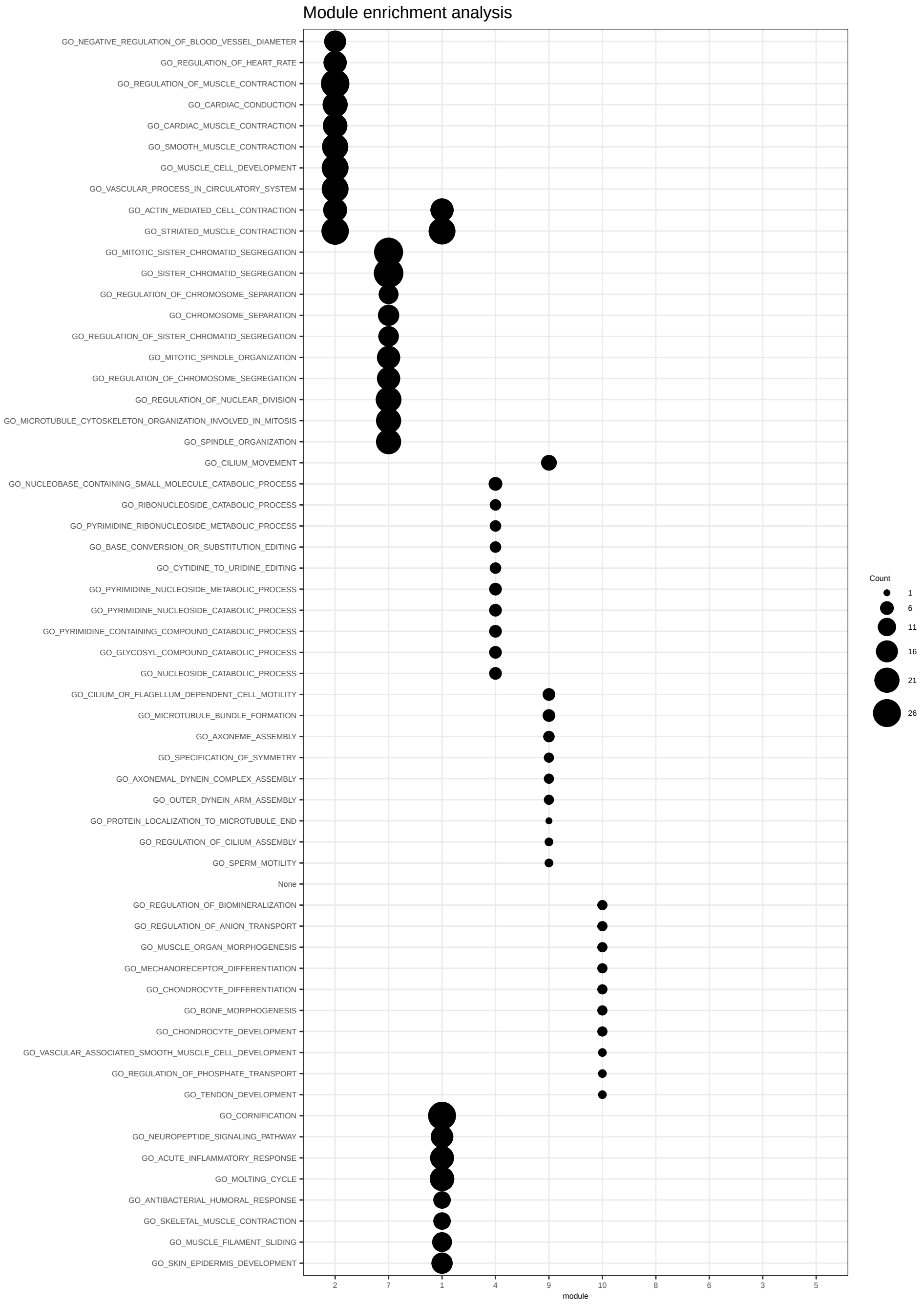

Figure S15: PRAD WGCNA module GO GSEA.

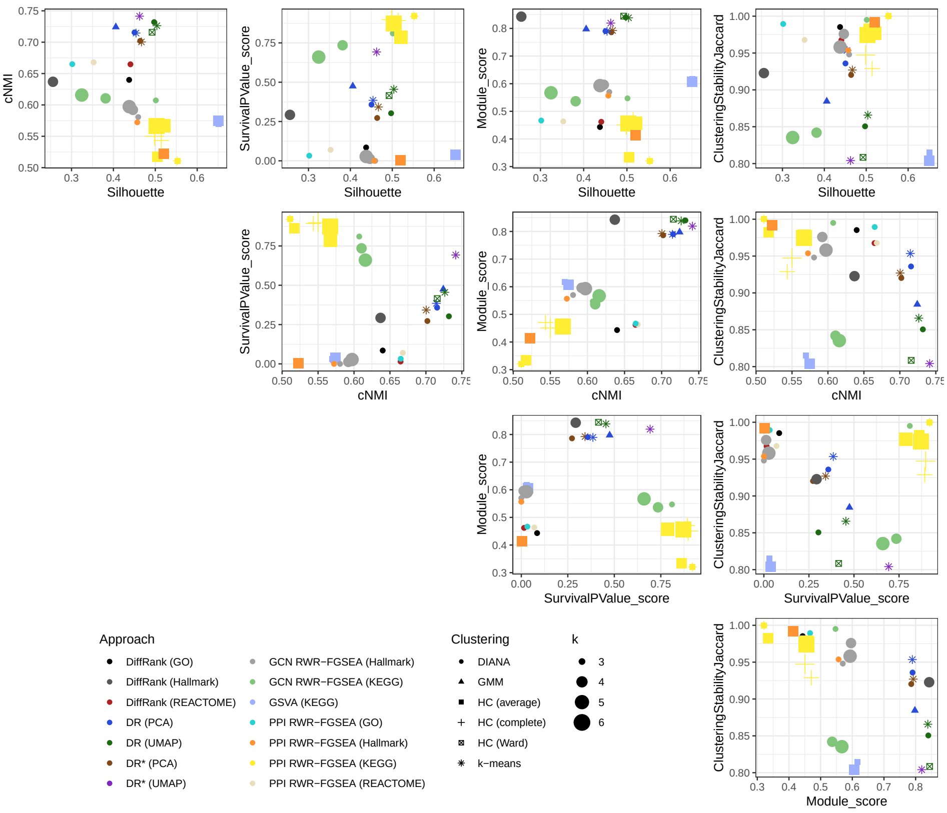

Figure S16: First Pareto frontier of clustering results based on several evaluation metrics. All values are averages from 100 resampled data subsets. DR* corresponds to dimensionality reduction that was applied on all genes i.e. without feature selection.

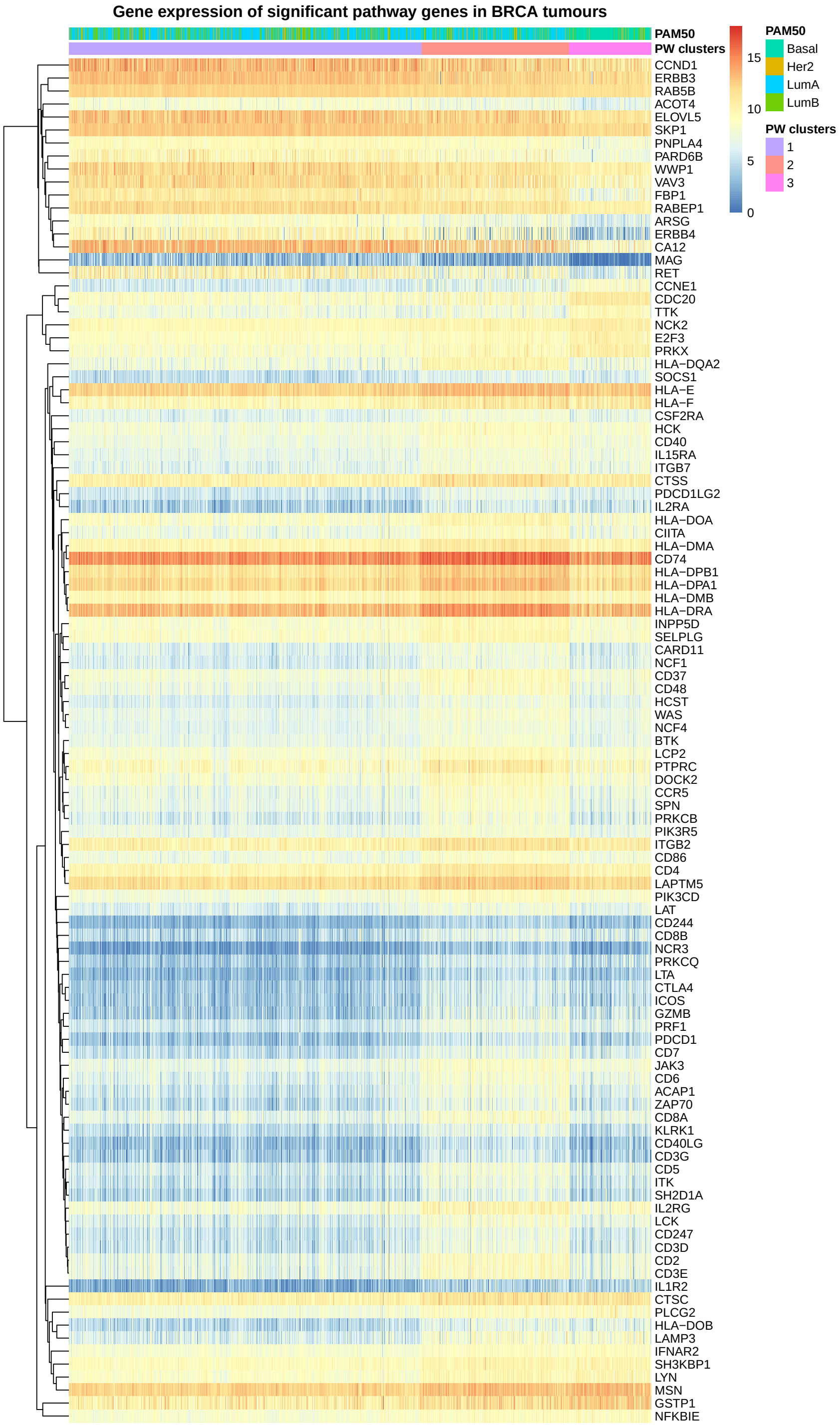

Figure S17: Gene-expression ($\log_2$ of TPM) of most informative pathway genes with significantly different expression between clusters in the best result (Kruskal-Wallis test p-value $< 10^{-50}$) for BRCA.

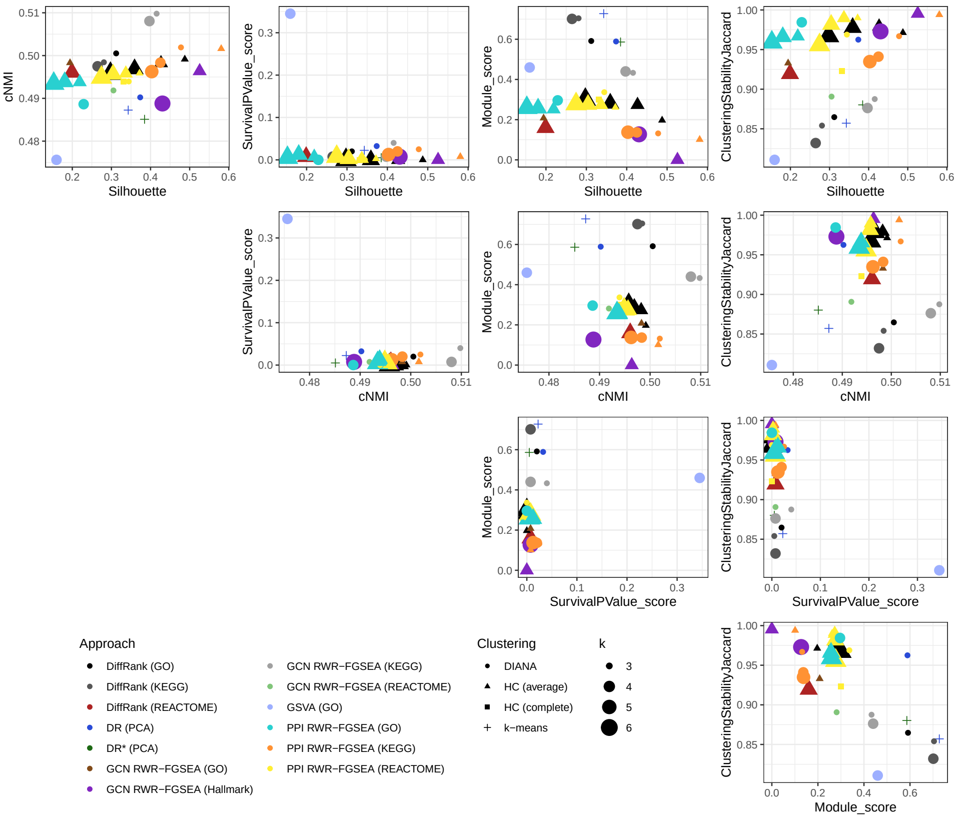

Figure S18: First Pareto frontier of PRAD clustering results evaluated by silhouette score, clustering stability, cNMI, survival significance and module score. All values are averages from 100 resampled data subsets. DR* corresponds to dimensionality reduction that was applied on all genes i.e. without feature selection.

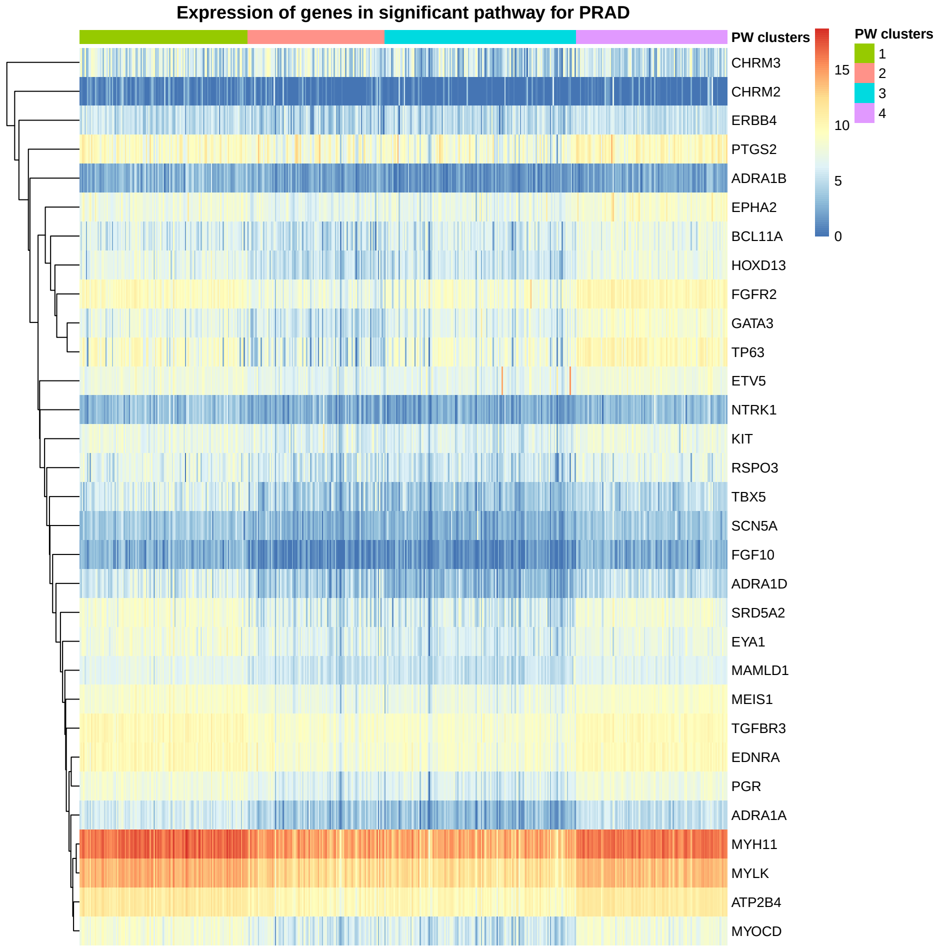

Figure S19: Gene-expression ($\log_2$ of TPM) of most informative pathway genes with significantly different expression between clusters in the best result (Kruskal-Wallis test p-value $< 10^{-50}$) for PRAD.

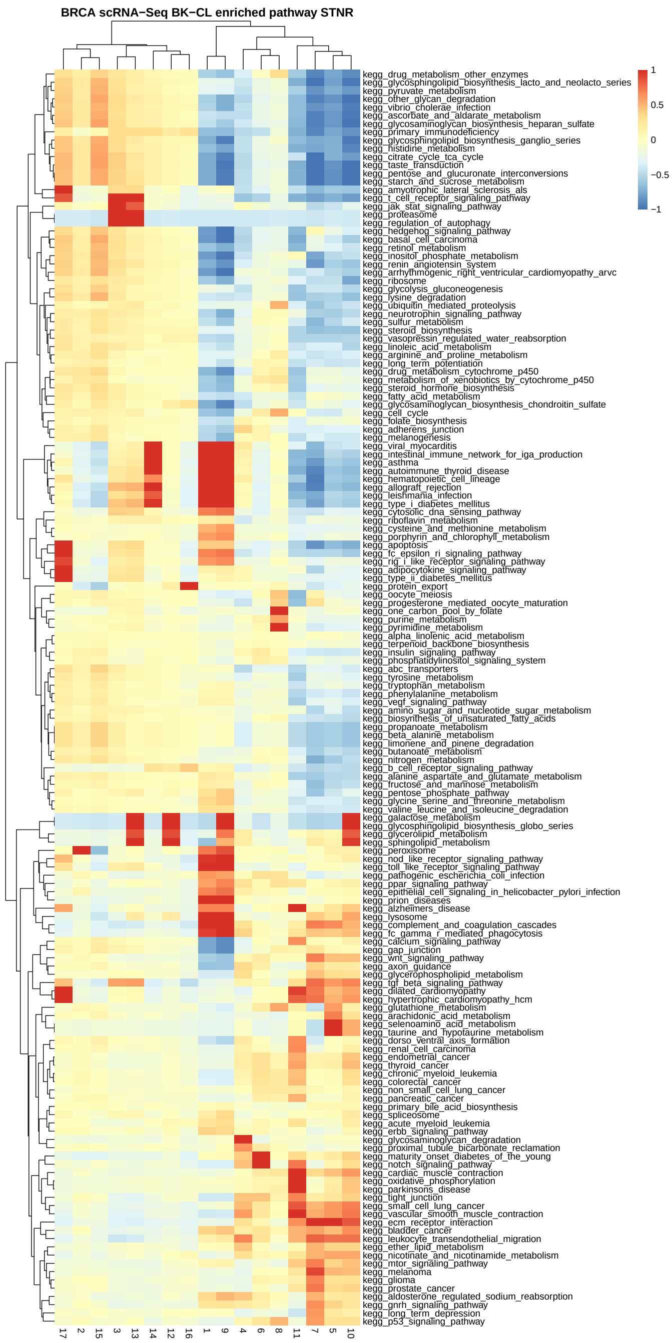

Figure S20: A heatmap of signal to noise ratio of BK-CL clusters and related KEGG-pathway-markers that were compiled to characterize single-cell RNA sequencing (scRNA-seq) profiles. The DiffRank-KEGG-Kmeans approach was tested on scRNA-seq data retrieved from the GEO database (GSE176078). This dataset refers to 26 primary tumors from three major clinical subtypes of breast cancer, including 11 ER+, 5 HER2+ and 10 TNBC. The values were truncated to [-1,1] for better contrast.

Table S1: Summary of data set samples. Clustering was applied to tumour samples while DEA was applied to tumour-control pairs and survival analysis was applied to patients with applicable data.

	Tumours	Controls	Survival data samples
BRCA	1041	112	979
PRAD	497	52	338

Table S2: Summary of gene filters. DEGs and disease associated genes from Open Targets were intersected to yield a set of highly relevant genes that were used as input to all methods. The set of all non-zero variance genes was also tested for the DR-CL approach for comparison.
[1] EFO_0000305 associated genes with genetic association score > 0.0 in Open Targets
[2] union of EFO_0000673 and EFO_0001663 associated genes with overall association score > 0.7 in Open Targets

	Non-zero variance genes	DEGs	OTP genes	DEGOTP genes
BRCA	19046	5036	549[1]	155
PRAD	19012	2988	961[2]	150

Table S3: BRCA Cox-PH covariate model Akaike information criterion (AIC) and Bayesian information criterion (BIC). The lowest p-value of the coefficients for each single variable model is also shown.

Variables	AIC	BIC	p-value
age	1343.79	1346.61	0.0000
stage	1321.05	1332.33	0.0009
pathology	1370.27	1381.55	0.0763
age + stage	1298.40	1312.50	
age + pathology	1348.08	1362.18	
stage + pathology	1322.81	1345.37	
age + stage + pathology	1300.19	1325.57	

Table S4: PRAD Cox-PH covariate model Akaike information criterion (AIC) and Bayesian information criterion (BIC). The lowest p-value of the coefficients for each single variable model is also shown.

Variables	AIC	BIC	pvalue
PSA	442.19	443.90	0.0441
age	441.93	443.64	0.0695
GC	426.16	431.30	0.9968
T	435.54	444.11	0.9963
N	407.57	409.24	0.0254
PSA + age	440.50	443.93	
PSA + N	408.59	411.92	
age + N	406.73	410.06	
PSA + age + N	407.18	412.17	

Table S5: Summary of methods used in the different clustering workflows. The DR-CL approach takes only gene expression as input, while BK-CL approaches also take pathway annotations and our novel network fusion approach takes a network describing interactions between genes in addition to the gene expression data and pathway annotations.

Input	Network analysis	Pathway enrichment	Low-dimensional embedding	Final dimensions	Distance metric	Clustering algorithms
Gene expression	-	-	PCA<br>t-SNE<br>UMAP<br>VAE	2-10	Euclidean	k-means<br>GMM<br>HC<br>diana
Gene expression<br>Annotations (GO, KEGG, REACTOME)	-	GSVA<br>DiffRank	-	N_{pathways}	Correlation	HC<br>diana
Gene expression<br>Annotations (GO, KEGG, REACTOME)<br>Networks (PPI, co-expression)	RWR from dysregulated seed genes	FGSEA	-	N_{pathways}	Correlation	HC<br>diana

Table S6: TCGA BRCA clinical variables in the best result analyzed in Figure 9. Age was divided into groups.
[1] invasive ductal carcinoma
[2] invasive lobular carcinoma

	Cluster	1	2	3	4
Stage	I	16	3	16	11
	II	64	51	67	59
	III	21	22	37	22
	IV	5	1	4	2
	(not defined)	1	6	0	6
Age	20-40	4	7	16	9
	40-50	21	12	23	24
	50-60	19	26	28	20
	60-70	35	25	37	28
	70-80	19	6	16	16
	80-100	9	7	4	3
PAM50 subtype	Basal	26	13	26	19
	Her2	8	5	9	8
	LumA	45	44	67	57
	LumB	28	21	22	16
Pathology	IDC[1]	56	39	45	39
	ILC[2]	8	11	18	21
	Mixed	11	9	9	7
	Other	13	5	4	11
	(not defined)	19	19	48	22

Table S7: TCGA PRAD clinical variables in the best result analyzed in Figure 9. Age was divided into groups. The median, first and third quartile as well as 2.5% and 97.5% quantile of pre-operation PSA in each cluster is shown. Mutation based subtypes identified by [9].

	Cluster	1	2	3	4
N-stage	n0	69	44	64	63
	n1	5	15	20	16
	(not defined)	19	7	7	9
T-stage	t2a	0	3	0	2
	t2b	1	1	1	1
	t2c	42	19	20	29
	t3a	34	25	31	26
	t3b	13	17	35	28
	t4	2	1	3	0
	(not defined)	1	0	1	2
Age	40-50	12	4	2	3
	50-60	36	27	30	33
	60-70	39	32	49	46
	70-80	6	3	10	6
Gleason	<7	14	6	5	6
	>7	20	32	49	37
	3+4	38	17	17	27
	4+3	21	11	20	18
PSA	Q0.025	3.00	2.33	2.28	2.77
	Q0.25	4.80	5.52	5.85	4.97
	Q0.5	7.00	7.55	8.00	7.20
	Q0.75	10.8	14.6	11.8	10.2
	Q0.975	42.9	42.9	29.8	28.3
Mutation Subtype	1.ERG	34	23	28	24
	2.ETV1	2	6	6	8
	3.ETV4	5	0	4	2
	4.FLI1	2	0	0	1
	5.SPOP	3	4	7	7
	6.FOXA1	1	2	2	0
	7.IDH1	2	0	0	0
	8.other	21	12	10	14
	(not defined)	23	19	34	32

References

[1] Diederik P Kingma and Max Welling. Auto-encoding variational bayes. *arXiv*, dec 2013.

[2] Kaiming He, Xiangyu Zhang, Shaoqing Ren, and Jian Sun. Delving deep into rectifiers: Surpassing human-level performance on ImageNet classification. In *2015 IEEE International Conference on Computer Vision (ICCV)*, pages 1026–1034. IEEE, dec 2015.

[3] Diederik P. Kingma and Jimmy Ba. Adam: A method for stochastic optimization. *arXiv*, dec 2014.

[4] Vladimir Yu Kiselev, Kristina Kirschner, Michael T Schaub, et al. SC3: consensus clustering of single-cell RNA-seq data. *Nature Methods*, 14(5):483–486, may 2017.

[5] Christopher R John, David Watson, Michael R Barnes, et al. Spectrum: fast density-aware spectral clustering for single and multi-omic data. *Bioinformatics*, 36(4):1159–1166, feb 2020.

[6] Vincent D Blondel, Jean-Loup Guillaume, Renaud Lambiotte, and Etienne Lefebvre. Fast unfolding of communities in large networks. *Journal of Statistical Mechanics: Theory and Experiment*, 2008(10):P10008, oct 2008.

[7] Jacob H Levine, Erin F Simonds, Sean C Bendall, et al. Data-driven phenotypic dissection of AML reveals progenitor-like cells that correlate with prognosis. *Cell*, 162(1):184–197, jul 2015.

[8] Patrick Roocks. Computing pareto frontiers and database preferences with the rPref package. *The R journal*, 8(2):393, 2016.

[9] Cancer Genome Atlas Research Network. The molecular taxonomy of primary prostate cancer. *Cell*, 163(4):1011–1025, nov 2015.
